# Supplementary material for: AI co-pilot bronchoscope robot
Source: Nat Commun. 2024 Jan 4;15:241. doi: 10.1038/s41467-023-44385-7 (PMC10764930; doi:10.1038/s41467-023-44385-7)
Supplement: Supplementary file 1 — Supplementary Information [file 41467_2023_44385_MOESM1_ESM.pdf]

Supplementary Information for

AI Co-pilot Bronchoscope Robot

Jingyu Zhang, Lilu Liu, Pingyu Xiang, Qin Fang, Xiuping Nie, Honghai Ma, Jian Hu, Rong Xiong*, Yue Wang*, Haojian Lu*

Correspondence to: luhaojian@zju.edu.cn; ywang24@zju.edu.cn; rxiong@zju.edu.cn

This PDF file includes:

Supplementary Note 1. Materials and fabrication of the bronchoscope robot.
Supplementary Note 2. Characteristic analysis of the bronchoscope robot.
Supplementary Note 3. Kinematic model and control strategy.
Supplementary Note 4. Establishment of virtual environment.
Supplementary Note 5. Datasets and comparison results of domain adaption.
Supplementary Note 6. Ending conditions in imitation learning.
Supplementary Note 7. Design and Fabrication of the breathing simulation system.
Supplementary Note 8. In-vitro phantom experiment.
Supplementary Note 9. Metrics.
Supplementary Note 10. NASA Task Load Index experiment.
Supplementary Note 11. AI intervention and safety ensure.
Supplementary Note 12. AI control performance analysis.
Supplementary Note 13. In-vivo animal experiment.
Supplementary Fig. 1. The CAD model of the AI co-pilot bronchoscope robot.
Supplementary Fig. 2. Characteristic analysis of the bronchoscope robot.
Supplementary Fig. 3. Kinematic model and control strategy.
Supplementary Fig. 4. Teleoperation experiment.
Supplementary Fig. 5. Virtual environment establishment and human command generation.
Supplementary Fig. 6. Simulation experiments in virtual bronchoscopy environments.
Supplementary Fig. 7. Simulation experiments on 10 different patients' airway models.
Supplementary Fig. 8. Image translation experiments of domain adaptation methods.
Supplementary Fig. 9. Training of structure-preserving unpaired image translation network.
Supplementary Fig. 10. Design and analysis of the breathing simulation system
Supplementary Fig. 11. Images on a specified path obtained by the paticipents.
Supplementary Fig. 12. Actuation information on a specified path obtained by the paticipents.
Supplementary Fig. 13. 3D trajectories on a specified path obtained by the paticipents.
Supplementary Fig. 14. Images on multiple paths obtained by the paticipents.
Supplementary Fig. 15. Actuation information on multiple paths obtained by the paticipents.
Supplementary Fig. 16. 3D trajectories on multiple paths obtained by the paticipents.
Supplementary Fig. 17. Reference paths and criteria of successful path.
Supplementary Fig. 18. Image error to 3D position error mapping.
Supplementary Fig. 19. Multi-dimensional reports with and without AI co-pilot acquired by NASA-TLX questionnaires.
Supplementary Fig. 20. Visualization of AI co-pilot intervention on human control.
Supplementary Fig. 21. Software-controlled GUI interface.
Supplementary Fig. 22. Closed-loop control experiment of the AI co-pilot bronchoscope robot.
Supplementary Fig. 23. Visualization of steering actions and tendon displacements during bronchoscopy.

Supplementary Fig. 24. 3D trajectories of in-vivo experiments.
Supplementary Fig. 25. Gradient-weighted Class activation maps (GradCAM) from the last convolutional layer of our policy network.
Supplementary Table 1. The operation experience of participants in the experiments.
Supplementary Table 2. Robot Costing.
Supplementary Table 3. Network architecture of our domain adaptation method.
Supplementary Table 4. Policy network architecture.

Supplementary Notes

Supplementary Note 1. Materials and fabrication of the bronchoscope robot

Supplementary Fig. 1 offers the CAD model of our AI co-pilot bronchoscope robot, designed to incorporate the plug-and-play bronchoscope catheters. The catheter is fixed on the guide seat, driven by two pairs of antagonistic tendons to achieve omnidirectional bending deformation of the distal section. The tendons are divided into four directions along the grooves of the guide seat and connect with the upper magnet holders. The steering control system consists of four linear motors (LA50-021D, Inspire-Robots, Beijing, China) for pulling the tendons and four force sensors (QLA414, FUTEK, California, America) for measuring the driving force. One side of each force sensor is connected to the linear motor through the motor flange, and another side is connected to the lower magnetic holder. Two sets of magnets are respectively installed inside the upper and lower magnet holders. Based on the magnetic adsorption force, the bronchoscope catheter can be quickly installed on the steering control system. The linear motors are installed on the motor fixture which connects with the electric slide (EZSM3E040AZMK, Oriental Motor, Tokyo, Japan) by the mounting shell to achieve the feed movement of the bronchoscope catheter. The electric slide is actuated by the slide driver (AZD-KD, Oriental Motor, Tokyo, Japan), and installed on the robotic arm (UR5, Universal Robots, Odense, Denmark) to achieve the large range of pose adjustment of the bronchoscope robot.

The bronchoscope catheter consists of the high-stiffness proximal section and the low-stiffness distal section. The proximal section uses the braided mesh structure for increased stiffness, while the distal section uses the snake tube made of stainless steel for steering control. The catheter is covered with the thin thermoplastic urethanes (TPU) layer for waterproofing. To improve the application range of the bronchoscope robot, a 3.3mm catheter with a 1.2mm working channel and a 2.1mm catheter are designed, enabling access to the ninth deeper generation bronchi for average adult patients. The two catheters are both installed with the micro camera (OCHTA10, OmniVision Technologies Inc., Carolina, America) with a square cross-section of 0.65*0.65mm, and two Led lights with a cross-section of 0.35*0.65mm. The proximal length of the two catheters is 650mm, while the snake bone length of the 3.3mm and 2.1mm catheter are respectively 35mm and 25mm. The distal section of the two catheters can achieve an omnidirectional bending of about 180 degrees for deep lung examination.

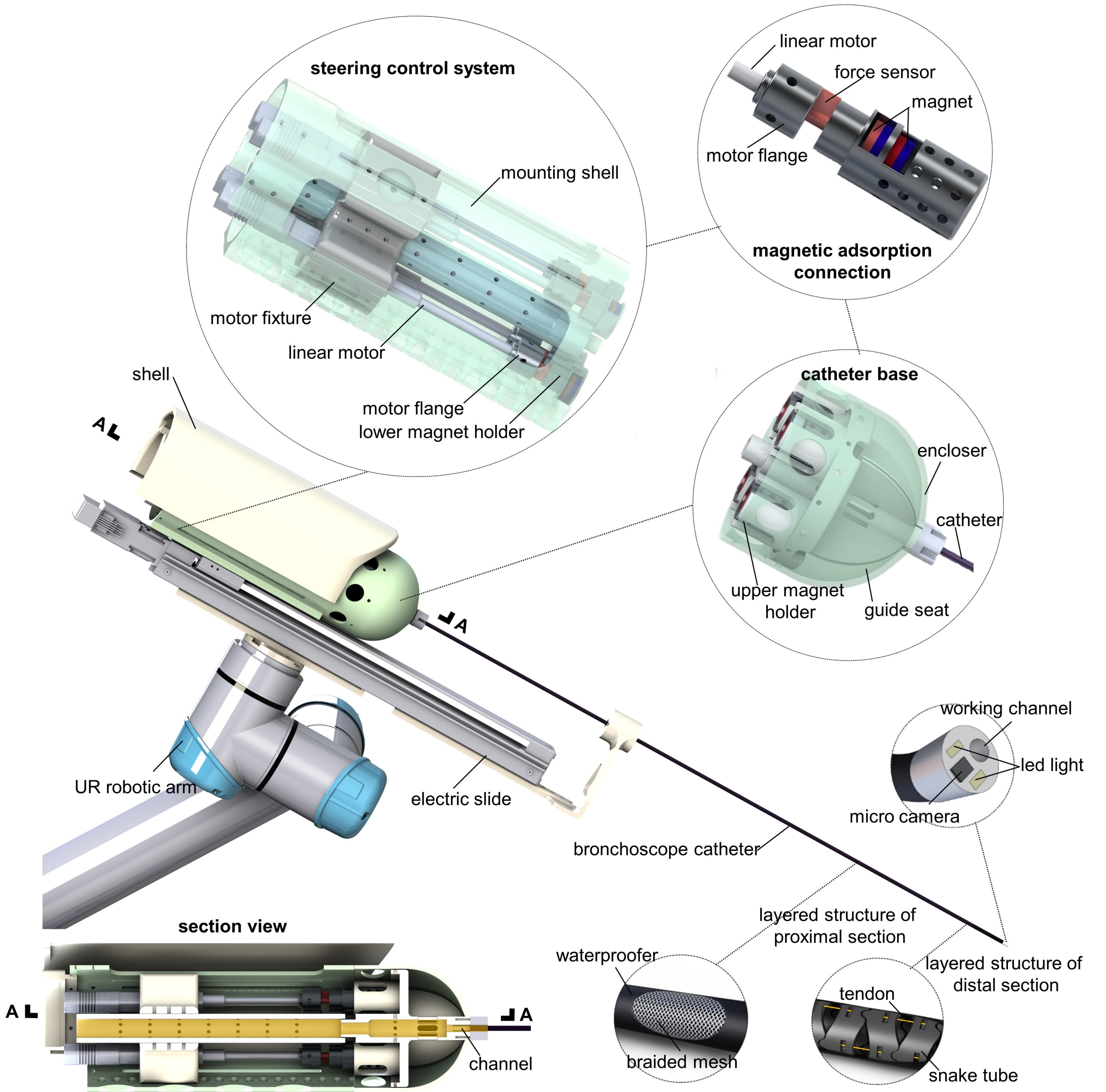

Supplementary Fig. 1. The CAD model of the AI co-pilot bronchoscope robot system. The system consists of a robotic arm, an electric slide, a steering control system, and plug-and-play bronchoscope catheters. The steering control system is composed of four linear motors to steer the catheter, and four force sensors to measure the actuation force. The steering control system and bronchoscope catheter are connected by magnetic adsorption force for rapid catheter replacement. The electric slide is used to feed the bronchoscope catheter, while the UR robotic arm is used to achieve the large range of pose adjustment of the bronchoscope robot.

Supplementary Note 2. Characteristic analysis of the bronchoscope robot

In order to comprehensively assess the performance of our bronchoscope catheters, a series of characterization experiments were meticulously conducted. Firstly, record the corresponding bending angle of each bronchoscope catheter alongside the actuation force when pulling the tendon. The results are illustrated in Supplementary Fig. 2a and 2b, where the bending angle and actuation force exhibit a linear increase with the increase of actuation displacement. The four tendons of each catheter have almost the same characteristics, but the two catheters have different rate of increase. This is mainly caused by the different mechanical and material parameters, such as the different elastic modulus and distances of the tendons from the centreline. Supplementary Fig. 2c shows the magnetic adsorption force of four groups of magnets employed in the connection between the steering control system and the bronchoscope catheter. The magnets adopted in our system have a diameter of 15mm and a height of 4mm, yielding the actual magnetic adsorption forces ranging between 6.7-7.5N, which demonstrates the force limit capacity of our system.

To reinforce safety, we implement soft limits on both actuation displacement and actuation force, simultaneously. Supplementary Fig. 2d illustrates the limitation factor heatmaps within a defined range of actuation force and displacement. Notably, the limitation factor indicates a safe distance from the limitation threshold. With the increase of actuation displacement and actuation force, the limitation factor becomes larger, meaning approaching the limitation threshold. This limitation factor serves as a crucial component in our control system, used for the adjustment of impedance stiffness of the teleoperator (Touch, 3D Systems, South Carolina, America). The lower the limitation factor, the smaller the impedance stiffness, which means that the operator can control the bronchoscope robot with ease.

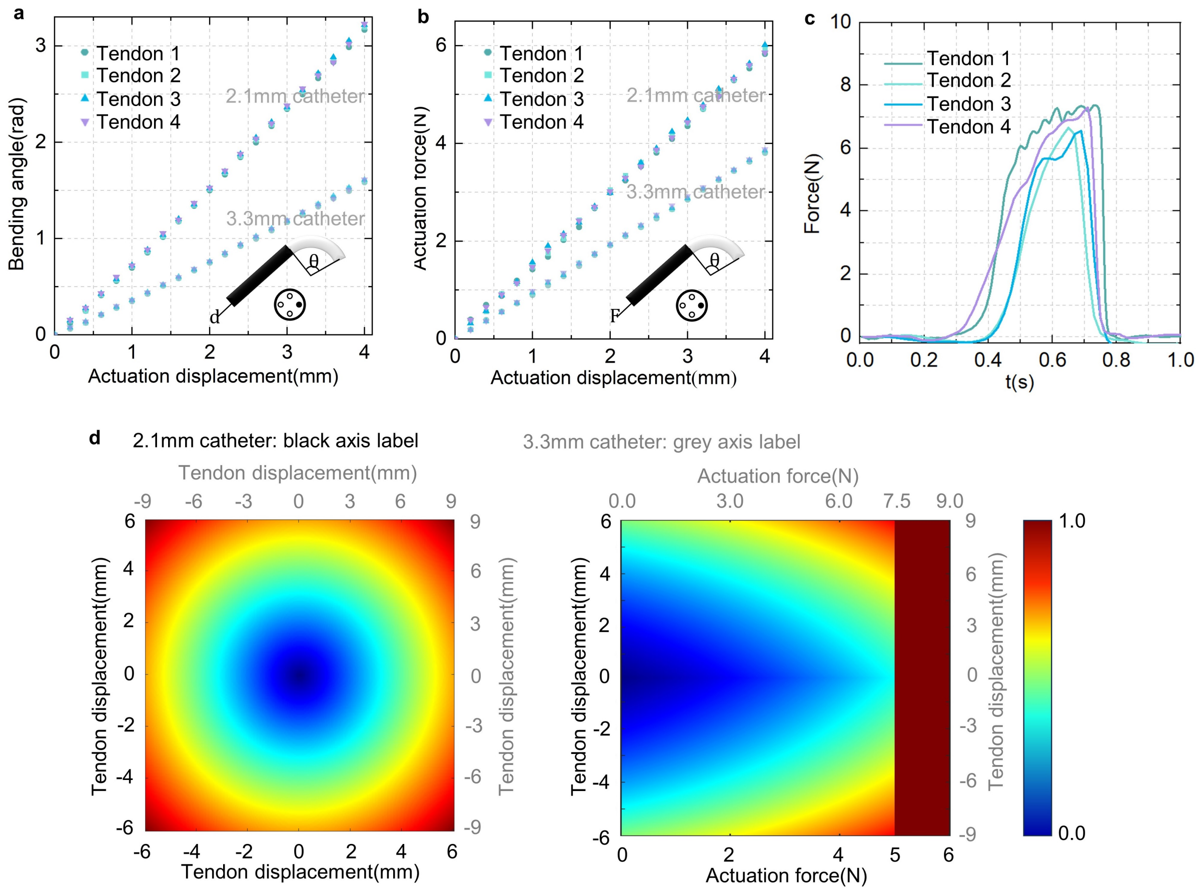

Supplementary Fig. 2. Characterization experiments of the bronchoscope robot. a, The relationship between the bending angle of distal section and actuation displacement. As the displacement increases, the bending angle increase linearly, and all four tendons of one catheter have almost the same characteristic. **b**, The relationship between the actuation force and actuation displacement. As the actuation displacement increases, the actuation force increases linearly, and all four tendons of one catheter have almost the same characteristic. **c**, The magnetic adsorption force of the magnets used in our system. The adsorption force is between 6.7-7.5N, which demonstrates the capability of force limit. **d**, The limitation factor heatmaps under a certain range of actuation force and actuation displacement.

Supplementary Note 3. Kinematic model and control strategy

Kinematic model: For simplifying the model, the following hypothesis is proposed. (1) The friction between the tendons and the catheter is ignored. (2) The tendons always run parallel to the centroidal axis of the catheter at a fixed distance. The coordinate system of the bronchoscope catheter is established shown in Supplementary Fig. 3a. The reference frame $\{o_b x_b y_b z_b\}$ and $\{o_p x_p y_p z_p\}$ are respectively established on the base and tip of the proximal section, and the reference frame $\{o_d x_d y_d z_d\}$ and $\{o_t x_t y_t z_t\}$ are respectively established on the tip of the distal section and the catheter tip. Their Z axes are perpendicular to the cross section of the catheter, whereas the X axes point to the first tendon, and Y axes are determined by the right-hand rule. The reference frame $\{o_c x_c y_c z_c\}$ of the micro camera is parallel to the frame $\{o_t x_t y_t z_t\}$. When the distal section deforms under the actuation of tendons, its configuration parameters can be represented by the bending angle θ, bending plane angle φ and the length l. The transformation relationship between $\{o_b x_b y_b z_b\}$ and $\{o_t x_t y_t z_t\}$ can be expressed as

$$\mathbf{T_b^t} = \mathbf{T_b^p} \cdot \mathbf{T_p^d}(\theta, \varphi, l) \cdot \mathbf{T_d^t} \tag{1}$$

where

$$\mathbf{T_p^d}(\theta, \varphi, l) = \begin{bmatrix} \cos^2\varphi(\cos\theta - 1) + 1 & \sin\varphi\cos\varphi(\cos\theta - 1) & \cos\varphi\sin\theta & l\frac{\cos\varphi(1-\cos\theta)}{\theta} \\ \sin\phi\cos\varphi(\cos\theta - 1) & \cos^2\varphi(1 - \cos\theta) + \cos\theta & \sin\varphi\sin\theta & l\frac{\sin\varphi(1-\cos\theta)}{\theta} \\ -\cos\varphi\sin\theta & -\sin\varphi\sin\theta & \cos\theta & l\frac{\sin\theta}{\theta} \\ 0 & 0 & 0 & 1 \end{bmatrix} \tag{2}$$

Define the actuation state **q** as follows:

$$\mathbf{q} = [l_1 \quad l_2 \quad l_3 \quad l_4]^T \tag{3}$$

where l_i represents the actuator displacement of the i-th tendon. The relationship between the actuation state and the configuration parameters could be represented as follows.

$$l_i = d\theta\cos(\alpha_i), i = 1, \dots, 4 \tag{4}$$

where d represents the distance between the tendon and the centre axis of the catheter, α_i is defined as

$$\alpha_i = \varphi - \pi\frac{i-1}{2}, i = 1, \dots, 4 \tag{5}$$

Teleoperation mapping relationship: In this work, the operator uses the teleoperator to control the bronchoscope robot. The teleoperator has six degrees of freedoms, more than 431*348*165mm workspace and two buttons. Under teleoperation control, the motion on the XY plane of the workspace of the teleoperator is used to control the omnidirectional bending of the catheter, while the two buttons are used to control the forward and backward movement

of the bronchoscope robot, as depicted in Supplementary Fig. 3b. Define the motion point on the XY plane of the workspace of the teleoperator as (x_t, y_t),

$$\theta = \theta_{\max}\sqrt{x_t^2 + y_t^2} \quad (6)$$

$$\varphi = \tan^{-1}\left(\frac{y_t}{x_t}\right) \quad (7)$$

where $\theta_{\max} = \pi$ is the maximum bending angle (180 degrees) of the distal section. During AI shared control, the XY plane of the workspace of the teleoperator are divided into five regions, corresponding to the discrete control commands (forward, up, down, left, and right).

Control method: As depicted in Supplementary Fig. 3c, there are two control modes: teleoperation control and AI shared control. During teleoperation control, the operator inputs the teleoperation command (motion trajectory of the teleoperator), then it is mapped into the configuration parameters of the bronchoscope catheter by equation (6-7). Furthermore, the configuration parameters are converted into the actuation displacements by equation (4-5). By the low-level controller, the linear motors can pull the tendons to steer the bronchoscope catheter towards the desired target. During AI shared control, the operator inputs the discrete command (forward, up, down, left, or right). Then, it is inputted into the AI shared control policy along with the bronchoscopic image. The policy outputs the configuration parameters of the bronchoscope catheter for the steering control, as mentioned before.

Teleoperation experiment: To test the performance of the teleoperation control, we hold the teleoperator moving along an approximate circle. During the process, the linear motors pull or push the tendons to steer the bronchoscope catheter by the proposed control strategy, as depicted in Supplementary Fig. 4a. Snapshots were taken of the catheter movement corresponding to the teleoperator's position at eight directions, as depicted in Supplementary Fig. 4b. The motion trajectory of the teleoperator in the motion process is depicted in Supplementary Fig. 4c, while the actuation displacements of two catheters converted from the trajectory is depicted in Supplementary Fig. 4d, e. The experiment fully verifies the performance of teleoperation control of the robot.

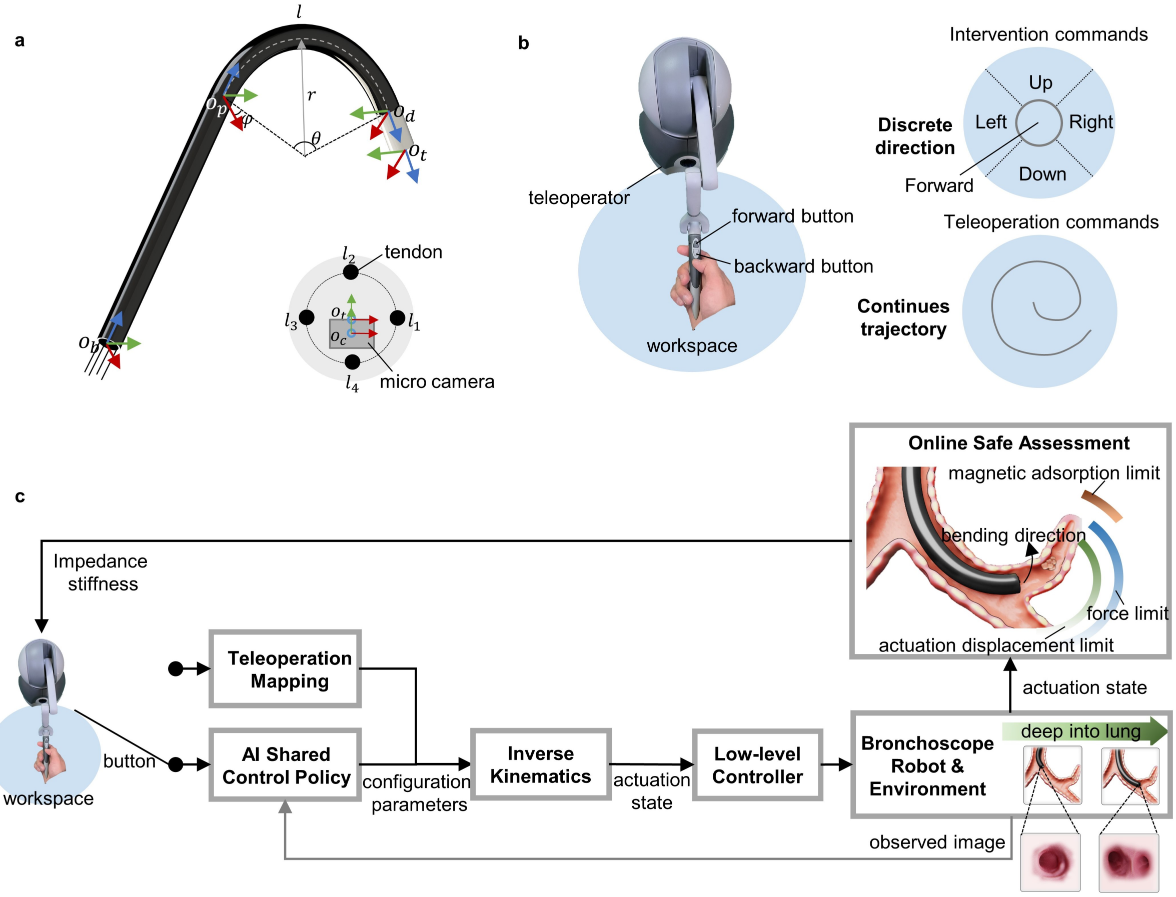

Supplementary Fig. 3. Kinematic model and control strategy. **a**, The kinematic schematic diagram of the bronchoscope catheter. o_b, o_p, o_d, o_t and o_c respectively represent the origin of the reference frame $\{o_b x_b y_b z_b\}$, $\{o_p x_p y_p z_p\}$, $\{o_d x_d y_d z_d\}$, $\{o_t x_t y_t z_t\}$ and $\{o_c x_c y_c z_c\}$. The configuration of the distal section can be represented by the bending angle θ, bending plane angle φ, and the length l. l_1, l_2, l_3 and l_4 represent the four tendon displacements. r is the arc radius. **b**, The teleoperation workspace of the teleoperator. During teleoperation control, the input command is continuous trajectory. During AI shared control, the input command is discrete directions. **c**, Control block diagram of the bronchoscope robot. The operator observes the images and inputs the teleoperation command, and then the command is converted to the configuration parameters of the distal section of the catheter. Next, the parameters are converted into actuation displacement by the inverse kinematics to control the steering of the bronchoscope catheter. The online safe assessment module adjusts the impedance stiffness of the teleoperator according to the limitation factor.

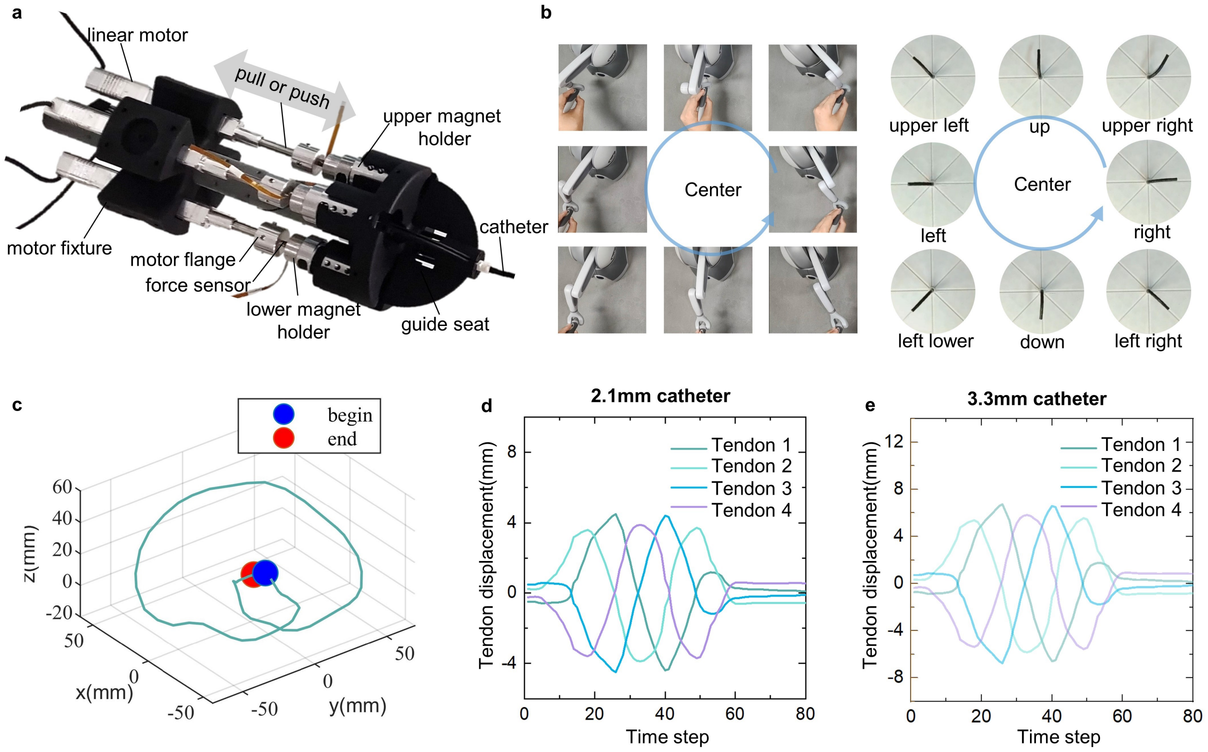

Supplementary Fig. 4. Teleoperation experiment. a, The inner structure of the bronchoscope robot. The linear motors pull or push the tendons to steer the bronchoscope catheter. **b**, Snapshots of the catheter deformation corresponding to the teleoperator's position at eight directions**. c**, The motion trajectory of the teleoperator in its workspace. **d**, The actuation displacements of 2.1mm catheter converted from the motion trajectory of the teleoperator. **f**, The actuation displacements of 3.3mm catheter converted from the motion trajectory of the teleoperator.

Supplementary Note 4. Establishment of virtual environment

The establishment of the virtual bronchoscopy environment is depicted in Supplementary Fig. 5a. The airway model is segmented from the pre-operative thorax CT scans. To create the hollow airway model, suitable for texture mapping and rendering the inner face, the 3D airway mask is dilated in three directions: coronal, axial, and sagittal view. Subsequently, the original airway mask is subtracted from the dilated 3D mask to produce a hollow bronchus mask. This mask is then used for surface reconstruction, generating the hollow airway model. For clarity, the term 'airway model' will henceforth refer to the hollow airway model within this paper. For Sim-style image rendering, a pink texture is applied with an RGB color setting of (206, 108, 131) and metallic and roughness factors for the surface material are set at 0.1 and 0.7, respectively. For Real-style image rendering, which requires a more realistic texture, we utilize a historic clinical bronchoscopy video from pigs unrelated to our study. Six frames with clear visuals are selected, cropped, and replicated to create the realistic texture. In clinical scenarios, it's essential to keep the bronchoscope's head as centred as possible within the airway to prevent damaging or piercing the bronchial wall. As such, we define the safest trajectories as those that follow the airway centrelines, which are extracted from the airway model using VMTK. The trachea entrance denotes the start of the centreline, with the end located at the bronchus terminal. Each centreline undergoes a smoothing process and uniform sampling to ensure waypoints are equally spaced.

In the process of establishing virtual bronchoscopy environment, the airway segmentation from pre-operative CT volume is a key step for simulating bronchus and extracting centrelines as reference paths. In this study, we utilize the region-growing algorithm to segment airway tree from CT volume, by manually placing a seed within the trachea. The adjacent regions can be automatically annotated as the same label if the Hounsfield Unit (HU) values are similar. In practice, region-growing algorithm can be easily implemented by the Airway Segmentations Module of 3D Slicer.

Simulated bronchoscope robot: According to the design of real bronchoscope robot, we employ a virtual perspective camera and a spot light to simulate the robot's head, and they share the same position and orientation. We select Pyrender as the physical rendering engine. During data preparation of the training stage, the simulated bronchoscope robot is placed within the virtual environment, where images and depth can be observed by rendering from the airway model.

The pose of simulated robot's head is identical to that of the camera, represented by the rotation and translation. The rotation matrix can be represented by three Euler angles: pitch,

yaw and roll, following an "XYZ" sequence. In the camera coordinate system $\mathbf{O_c}$ of the robot, pitch, yaw and roll rotations correspond to movements around the side-to-side (x axis), vertical axes (y axis) and front-to-back (z axis), respectively. As shown in Supplementary Fig. 5b, the roll rotation of robot is fixed due to mechanical constraints in practice, so we set the rate of roll to 0 and use (Δyaw, Δpitch, 0), i.e. ($\Delta\theta$, $\Delta\varphi$, 0), to represent the angle rate as a steering action in current $\mathbf{O_c}$ that rotates the robot's head towards the next location. Then a forward action is needed to move the robot to reach that position. For safety and maneuverability, we assign a fixed forward speed to the robot. Clinicians can adjust or halt this speed at any time in practice. In clinical scenarios, the skillful control of rotation angle rates to evade contact with the bronchial wall requires considerable expertise. Therefore, we address this challenge by introducing an AI-human shared control algorithm in this paper. This algorithm automatically generates safe $\Delta\theta$ and $\Delta\varphi$ angles based on human commands, enabling the bronchoscope robot to follow the optimal safe path, i.e. the airway centreline.

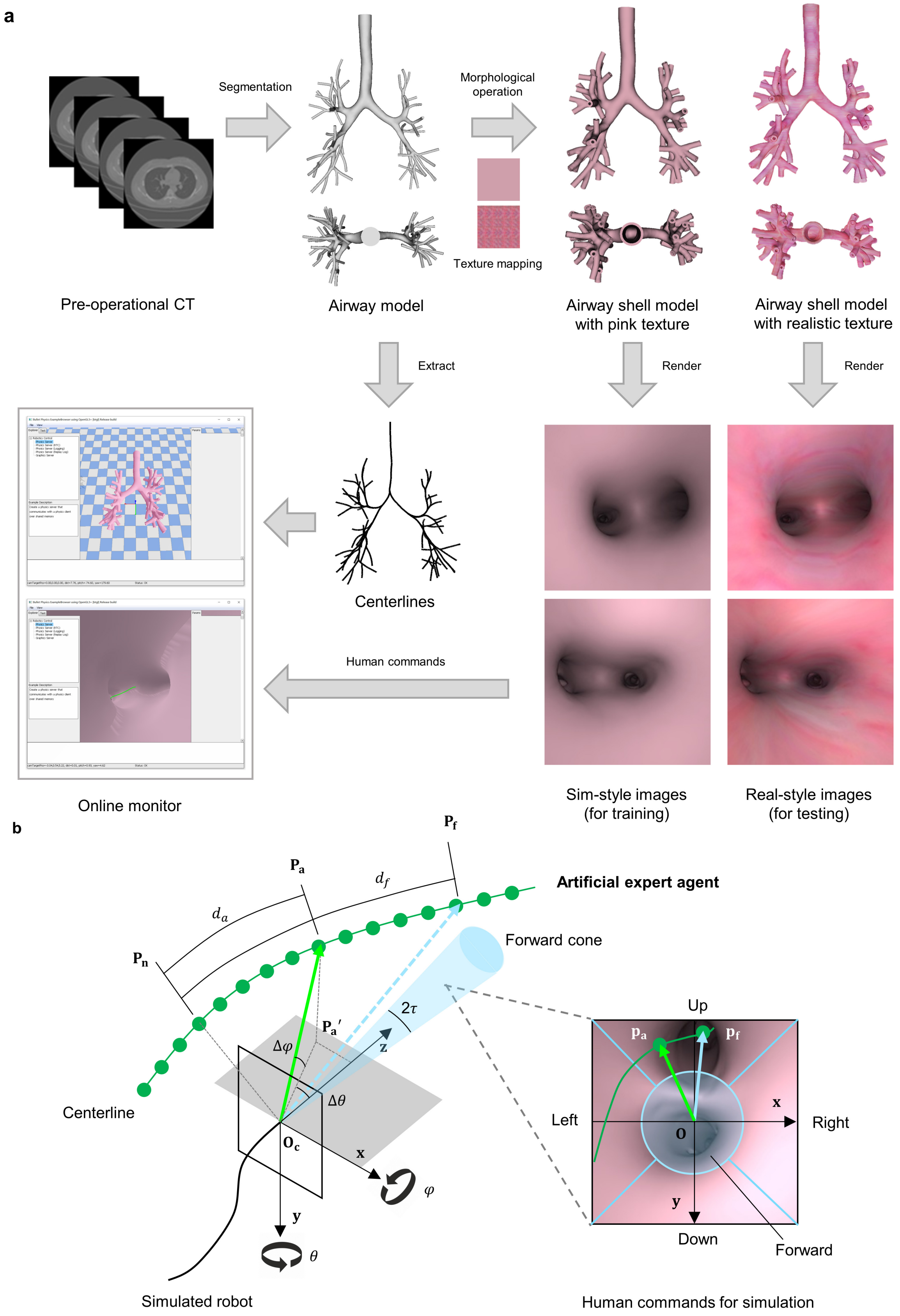

Supplementary Fig. 5. Virtual environment establishment and human command generation. a, Establishment of virtual bronchoscopy environment. A per-operational CT is acquired and the airway is segmented to generate an airway model. Centrelines of the airway model are extracted by VMTK tool as the ground truth safe trajectories for training. Next, an

airway shell model is generated by dilation and subtraction operation, with pink and realistic texture mapping respectively. The observation of the simulated bronchoscope robot is rendered from the bronchus shell model using Pyrender. In policy training stage, Sim-style bronchoscopic images are used for training and Real-style for testing. For online monitoring of the training process, all models are loaded in PyBullet environment for visualization and interaction. **b**, Illustration of AEA. The human commands and ground truth steering actions are automatically generated by AEA rather than doctors in the training stage of policy network, alleviating the burden of human annotation.

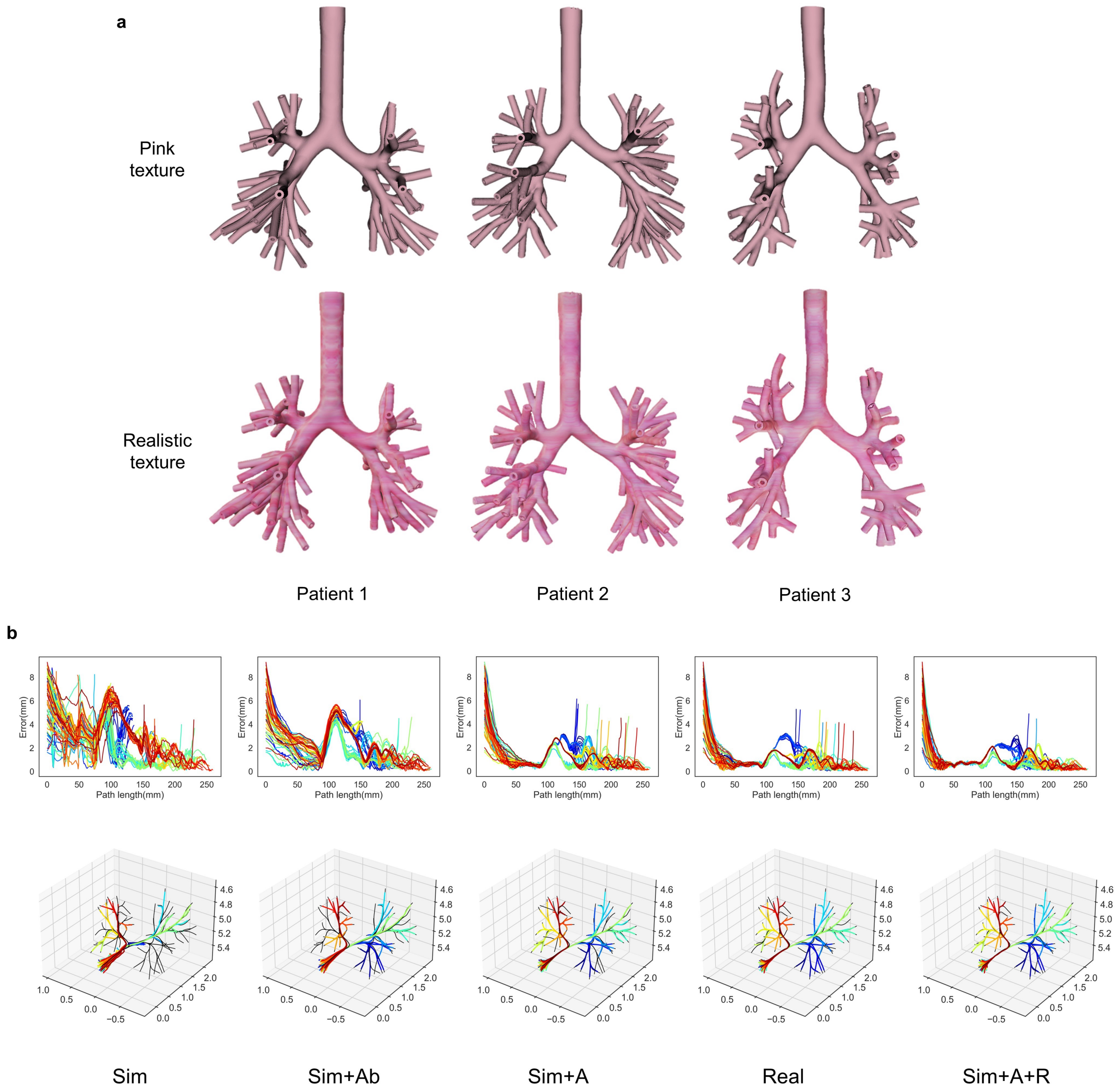

Supplementary Fig. 6. Simulation experiments in virtual bronchoscopy environments. a, Three airway models containing up to 5th generation of bronchi from three patients. The upper models are mapped with pink texture and the lower models are mapped with realistic texture. In our study, Patient 1 and Patient 2 with pink texture are used for training the policy network and the Patient 3 with realistic texture is used for testing. **b**, Specific trajectory errors and 3D trajectories of 60 paths of different methods tested in patient 3. Colored lines represent predicted trajectories and the black lines represent the reference paths. It's shown that training in virtual environment by using Sim-style images and adding our domain adaptation and randomization method (Sim+A+R) can achieve the highest accuracy and success rate.

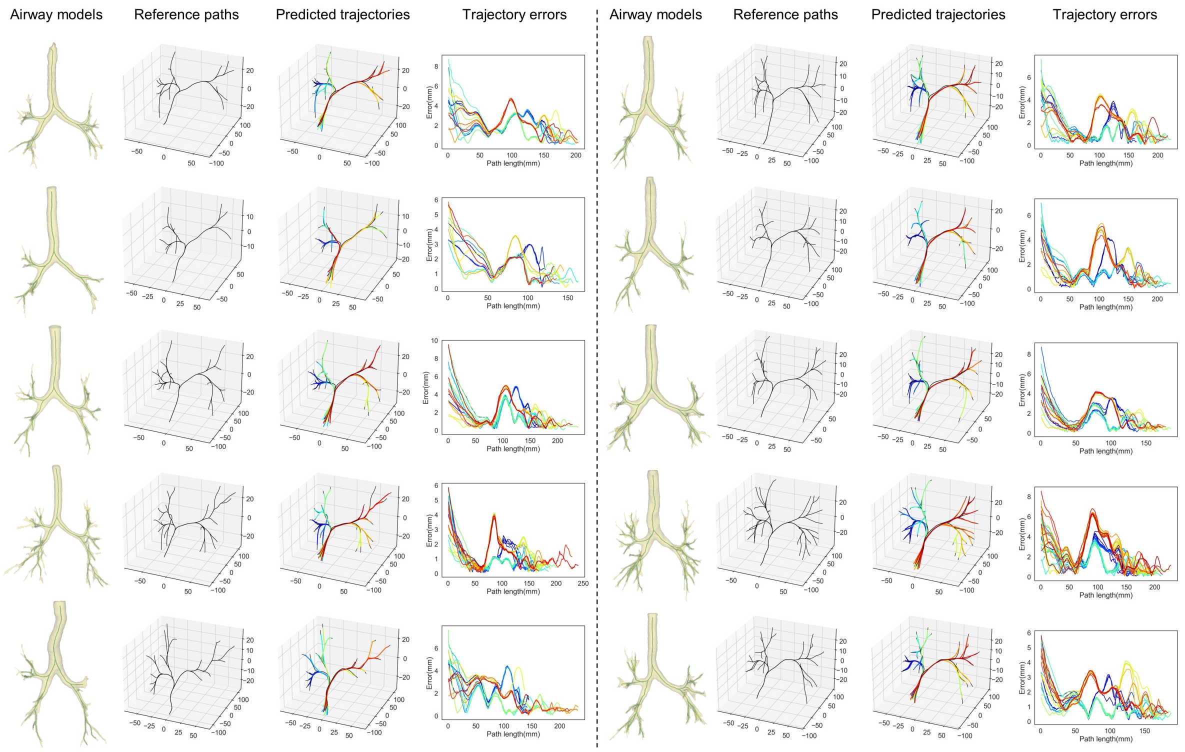

Supplementary Fig. 7. Simulation experiments on 10 different patients' airway models. Ten airway models of ten patients are segmented from CTs by using region growing method in 3D Slicer. These airway models contain up to 4th or 5th generation of bronchi. By testing our Sim+A+R method in these airway models with realistic texture mapping, the predicted 3D trajectories and corresponding reference paths of each patient are recorded and the trajectory errors are computed. 192/194 paths are successfully completed by our policy network, reaching a mean ± SD at 1.81±1.29mm. It's shown that our method is robust to different bronchial structures and segmentation qualities of airway models and can be successfully generalized to unseen patients.

Supplementary Note 5. Datasets and comparison results of domain adaption

To evaluate the effectiveness of the proposed domain adaptation method in image translation, three unpaired realistic datasets with different styles are introduced as target domains for training and evaluation. The source domain consists of Sim-style images rendered from CT airway model with pink texture. As shown in Supplementary Fig. 8a, three target domains are respectively rendered images from CT airway model with realistic texture (Real-style), real bronchoscopic images in silicone phantom (Phantom-style) and real historical bronchoscopic images in pigs (Clinical-style). As comparison, original AttentionGAN is implemented as the baseline for image translation from source to target domain. The Supplementary Fig. 8b shows that our method can effectively perform image translation while preserving bronchial structures, but the baseline method mistakenly considers the bronchial structure as a part of image style to translate and fails to preserve bronchial structures after image translation.

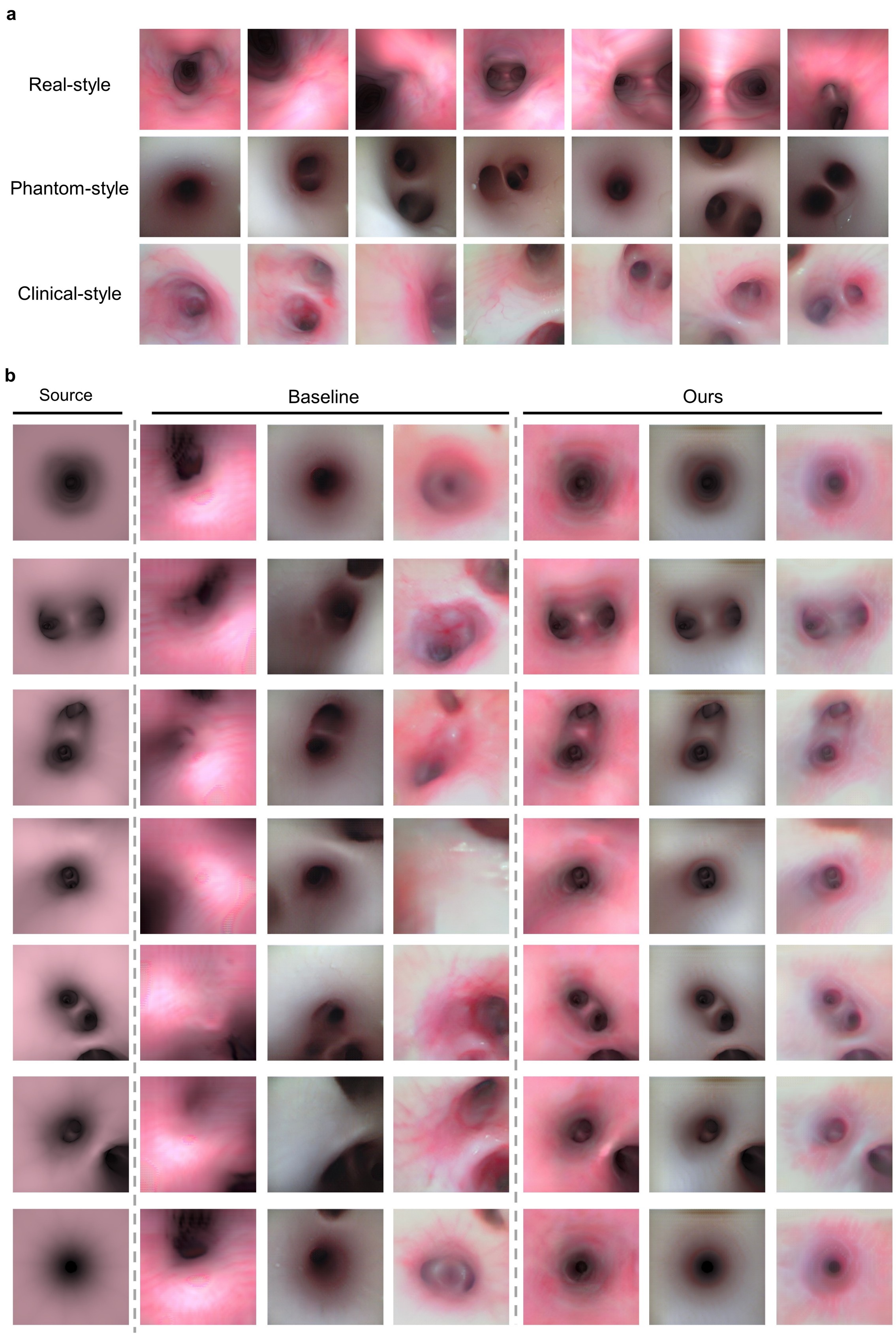

Supplementary Fig. 8. Image translation experiments of domain adaptation methods. a, Three unpaired realistic datasets with different styles as target domains for training and evaluation. Real-style images are rendered from CT airway model with realistic texture. Phantom-style images are collected from real bronchoscopic videos in silicone phantom.

Clinical-style images are collected from historical real bronchoscopic videos in pigs. **b**, Qualitive results of image translation. Original AttentionGAN is implemented as the baseline for image translation from source to target domain. The results show that our method can effectively perform image translation while preserving bronchial structures, but the baseline method mistakenly considers the bronchial structure as a part of image style to translate and cannot preserve bronchial structures after image translation.

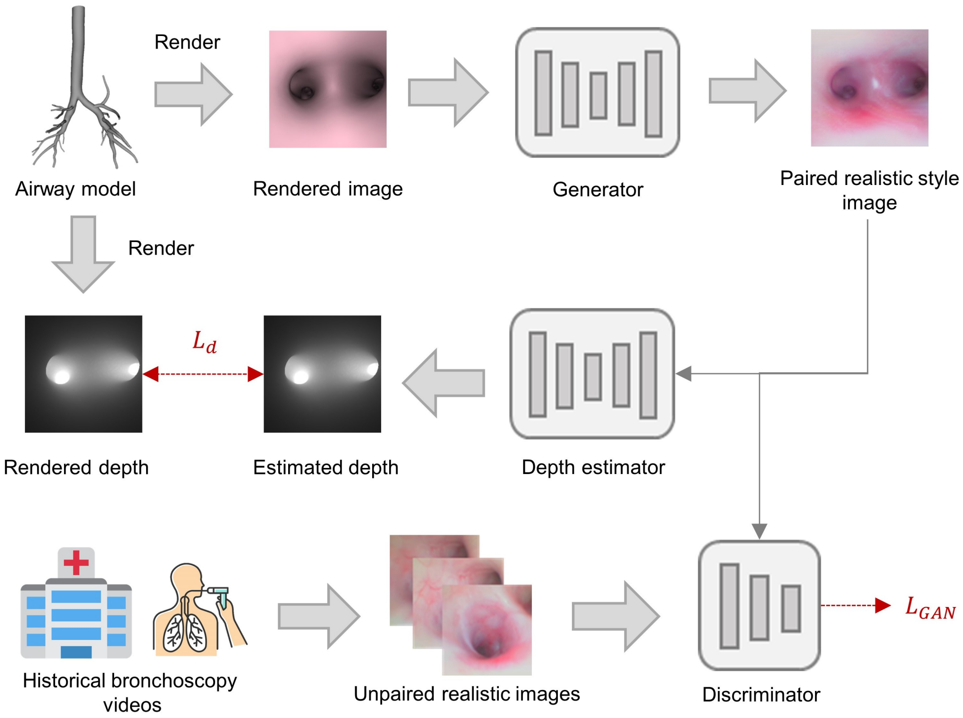

Supplementary Fig. 9. Training of structure-preserving unpaired image translation network. The network consists of a generator, discriminator and a depth estimator. The Sim-style images rendered from airway models with pink texture are collected as the source domain, and their corresponding depths are rendered to provide depth supervision. Unpaired clinical images from historical bronchoscopy videos serve as the target domain, which are easy to access in hospital. In the training stage, Sim-style images are fed into the generator to translate them into paired realistic-style images. Then the discriminator takes both translated realistic-style images and unpaired clinical images as input, forming the adversarial loss for training. Meanwhile, the realistic-style images are fed into the depth estimator for generating estimated depths, which are supervised by the rendered depths corresponding to the input rendered image, ensuring that the 3D structure information of the generated image remains consistent with the original rendered image. The "hospital icon" is designed by Twitter Emoji and is used under the open access CC BY 4.0 license (Creative Commons Attribution 4.0 International License).

Supplementary Note 6. Ending conditions in imitation learning

In on-policy training stage, the stopping conditions for each rollout include: reaching the endpoint, collision, exceeding the reference length, or exceeding the virtual channel. Specifically, (i) the run concludes when the bronchoscope robot reaches the ending point of the reference path. (ii) The run terminates if the robot collides with the environment. (iii) To avoid the situation that the untrained policy network $\boldsymbol{\pi}$ wanders in the environment for a long time and leads to overwhelming the whole dataset, the run ends when the total path length of the bronchoscope robot running in the environment is greater than the length of reference path. (iv) Since the ground truth action is calculated using the reference airway centreline, when the robot enters another airway unrelated to the current centreline, the derived ground truth action is wrong. Thus, we design a virtual channel for each centreline, and the run ends if robot exceeds the virtual channel.

Supplementary Note 7. Design and Fabrication of the breathing simulation system

The CAD model of the breathing simulation system is shown in Supplementary Fig. 10a. It consists of a crank and rocker mechanism to produce periodic reciprocating motion, a rotary motor along with the motor driver to actuate the crank. These components are both installed on the base. The crank, rocker, and other mounting parts are fabricated by 3D printed technology. The usage diagram is shown in Supplementary Fig. 10b. A bronchial phantom is installed on the breathing simulation system, and the bronchoscope robot is controlled deep into the phantom.

Motion analysis: To analyze the motion trajectory of the bronchial phantom on the breathing simulation system, the coordinate system is established shown in Supplementary Fig. 10c. Define the coordinates of points A, B, C, and D as (x_A, y_A), (x_B, y_B), (x_C, y_C) and (x_D, y_D), the rotation angles of three links as φ_1, φ_2 and φ_3, the length of three links as L_1, L_2 and L_3. Establish the vector equation as follows.

$$\overrightarrow{AB} + \overrightarrow{BC} = \overrightarrow{AD} + \overrightarrow{DC} \tag{8}$$

Establish the displacement equation as follows.

$$\begin{cases} x_B + L_2\cos\,\varphi_2 = x_D + L_3\cos\,\varphi_3 \\ y_B + L_2\sin\,\varphi_2 = y_D + L_3\sin\,\varphi_3 \end{cases} \tag{9}$$

The coordinate of B could be represented as

$$\begin{cases} x_B = L_1\cos\,\varphi_1 \\ y_B = L_1\sin\,\varphi_1 \end{cases} \tag{10}$$

Let tg $\beta = \frac{y_B - y_D}{x_B - x_D}$, we have

$$\cos\,(\beta - \varphi_3) = \frac{(x_B - x_D)^2 + (y_B - y_D)^2 + L_3^2 - L_2^2}{2L_3\sqrt{(x_B - x_D)^2 + (y_B - y_D)^2}} \tag{11}$$

$$\sin\,\varphi_2 = \frac{y_D + L_3\sin\,\varphi_3 - y_B}{L_2} \tag{12}$$

By the above equations, the motion trajectory of the breathing simulation system could be solved. The motion trajectory of the phantom in the experiment is shown in Supplementary Fig. 10d.

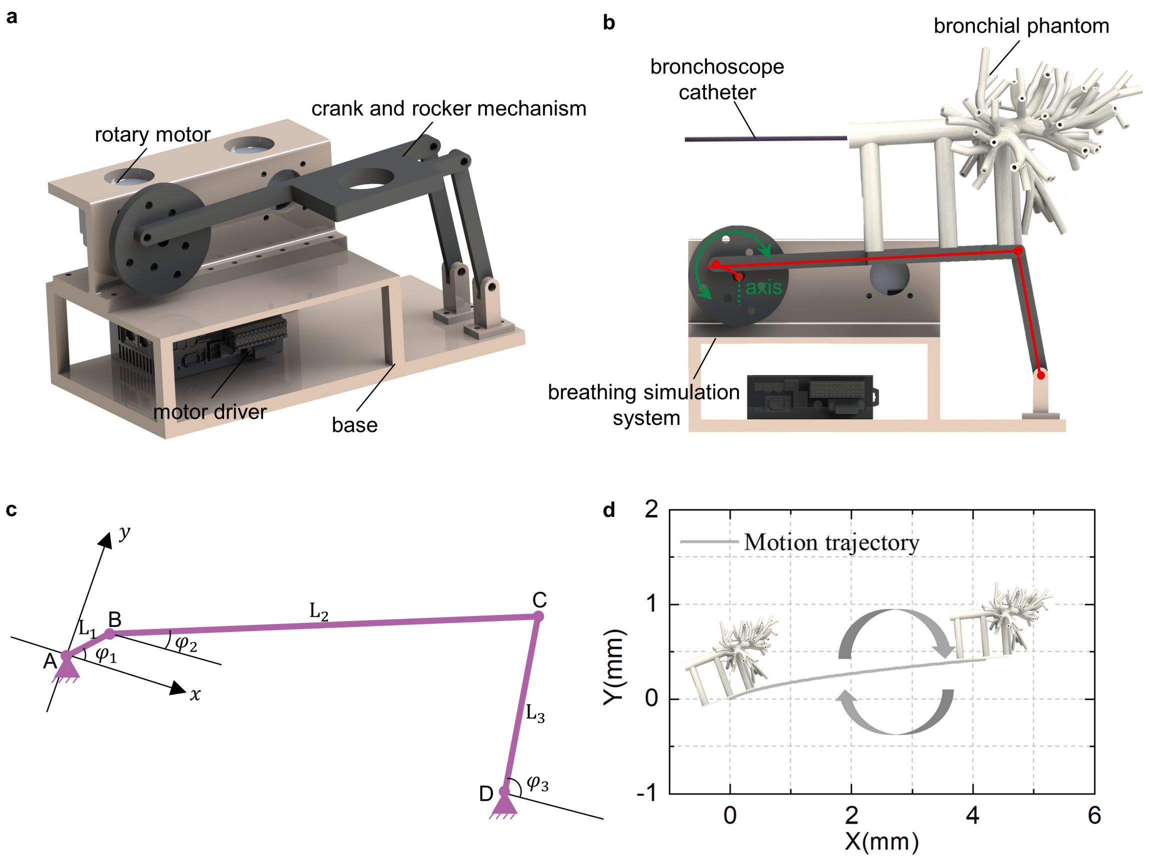

Supplementary Fig. 10. Design and analysis of the breathing simulation system. a, The CAD model of the breathing simulation system. It consists of a crank and rocker mechanism to produce periodic reciprocating motion, a rotary motor, and its motor driver to actuate the crank. **b**, The usage diagram of the breathing simulation system. **c**, The established coordinate system on the breathing simulation system. The rotation angles of three links are defined as φ_1, φ_2 and φ_3. The length of three links are defined as L_1, L_2 and L_3. **d**, The motion trajectory of the phantom in the in-vitro experiments.

Supplementary Note 8. In-vitro phantom experiment

Experimental setup: Three participants including two novice doctors and an expert took part in the experiment. The first novice doctor is a medical intern, and another novice doctor is a attending physician, both from the School of Medicine at Zhejiang University, Hangzhou, China. The expert is a chief physician, from the School of Medicine at Zhejiang University, Hangzhou,, China. The comparison of their operational experience is depicted in Supplementary Table 3. During the experiment, the bronchial phantom is placed on the crank-rocker mechanism-based breathing simulation system that emulates human respiration. The doctors hold the teleoperator to input the command for the steering control of the bronchoscope catheter.

In vitro experiment on a specified path: The first three groups of images of Supplementary Fig. 11 demonstrate the bronchoscopy process by the medical intern. It can be obverse that the bronchoscopy operation is difficult for a novice doctor without any experience. By the fourth group of images, even for the expert, it is difficult to keep the catheter tip centred all the time in the presence of fatigue or shaking hand. By the fifth group of images, AI co-pilot bronchoscopy operation has the best performance because the cavities are predominantly located at the centre of the image.

The actuation displacement curves and actuation force curves during intubation are plotted in Supplementary Fig. 12a and b. The curves obtained by the medical intern have increased minor fluctuations, which could be attributed to shaking hands. The expert operates with greater smoothness, showcasing performance akin to that of the attending doctor with AI co-pilot. It can be seen from Supplementary Fig. 12c and d that the AI co-pilot intubation has a reduced mean value and fluctuation range of actuation displacement and actuation force than the others. Supplementary Fig. 13a shows the specified path projected in the bronchial phantom, and the 3D trajectories in the five groups of trials are respectively depicted in Supplementary Fig. 13b, c, d, e and f. It is observed that the AI co-pilot bronchoscopy operation has the best effect, and the actual trajectory basically coincides with the reference trajectory. The 3D trajectory obtained by the medical intern has maximum operation error due to the lack of operational experience.

In vitro experiment on multiple paths: The two groups of images (Supplementary Fig. 14) demonstrate the bronchoscopy operation conducted by the expert and the attending doctor with AI co-pilot. It is observed that the bronchoscope robot could successfully navigate deep into the bronchial phantom along the desired paths, with the catheter tip centred. The result indicates the good control performance exhibited by both the expert and the attending doctor with AI co-pilot.

The actuation displacement curves and actuation force curves by the expert are respectively plotted in Supplementary Fig. 15a and b, while these by the attending doctor with AI co-pilot are plotted in Supplementary Fig. 15c, and d. Notably, the actuation displacement and force by AIco-pilot show reduced fluctuations in comparison to those by the expert. By Supplementary Fig. 15e and f, the AI co-pilot intubation demonstrates a smaller mean value and fluctuation range than teleoperation by the expert, which demonstrates that our AI co-pilot bronchoscope robot can equip a novice doctor with the skills to perform lung examination as expertly as a seasoned practitioner. Similarly, it can be seen from Supplementary Fig. 16 that the 3D trajectory obtained by the attending doctor with AI co-pilot has the best performance.

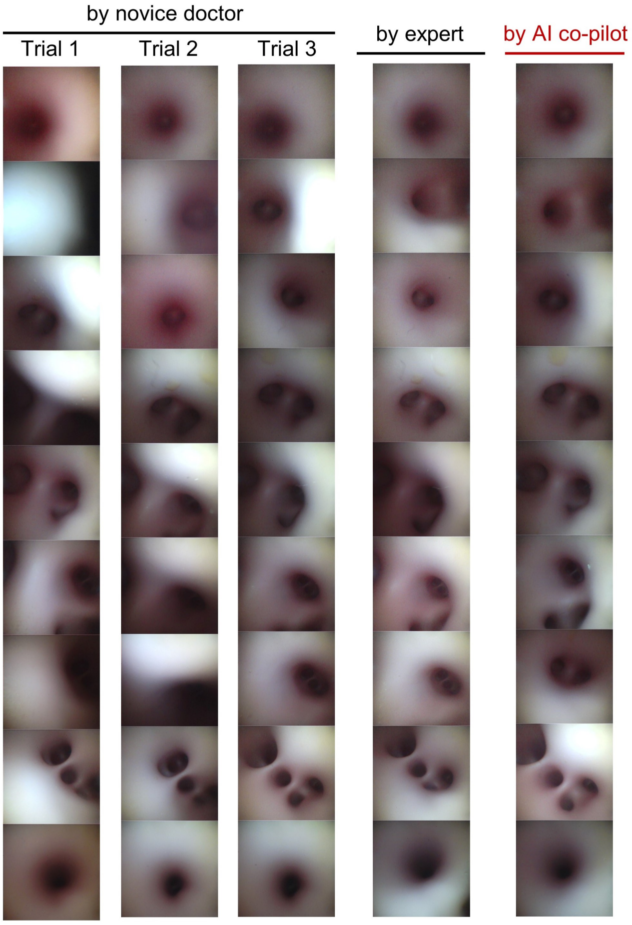

Supplementary Fig. 11. Images on a specified path obtained by the paticipents. It is obvious that bronchoscopy operation is difficult for the novice doctor. Even for the expert, bronchoscopy can be difficult in the presence of fatigue or hand shaking, making it challenging to prevent collisions. By comparison, AIco-pilot bronchoscopy has the best performance, and the cavities are predominantly located at the centre of the image.

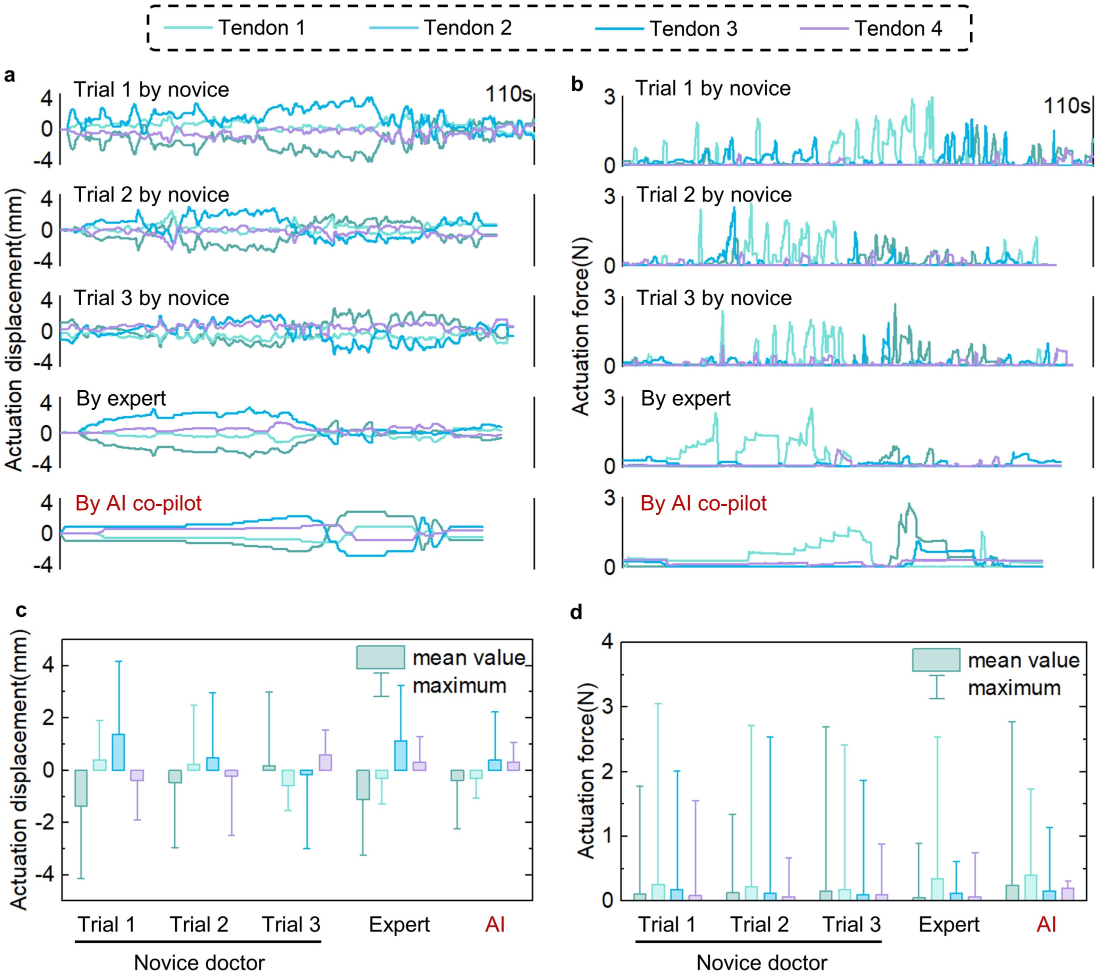

Supplementary Fig. 12. Actuation information on a specified path obtained by the paticipents. **a**, Actuation displacement curves obtained by the medical intern (the first three groups), the expert (the fourth group), and the attending doctor with AI co-pilot (the fifth group). **b**, Actuation force curves obtained by the medical intern (the first three groups), the expert (the fourth group), and the attending doctor with AI co-pilot (the fifth group). **c**, Distribution of actuation displacements. The numbers of recorded points are n = 4082, 3372, 3503, 3270 and 2558 for five independent experiments. **d**, Distribution of actuation forces. The numbers of recorded points are n = 4082, 3372, 3503, 3270 and 2558 for five independent experiments.

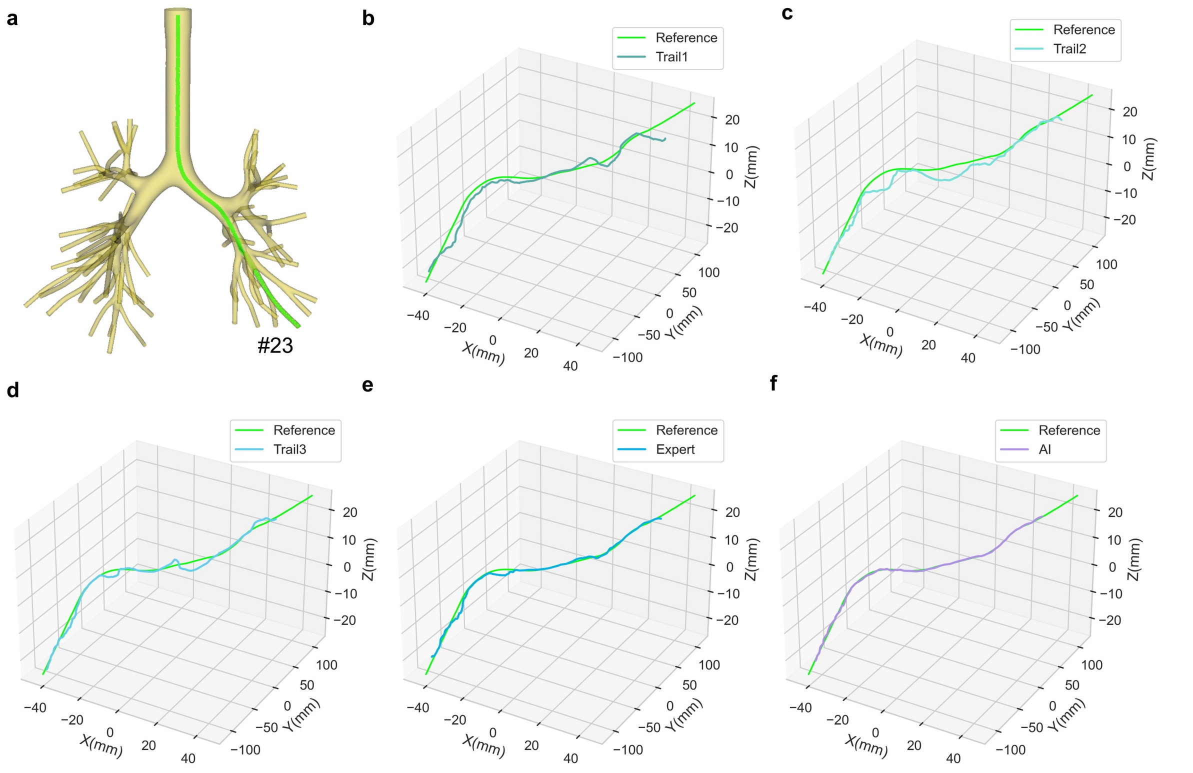

Supplementary Fig. 13. 3D trajectories on a specified path obtained by the participants. **a**, The airway model of phantom and the reference path chosen for performing bronchoscopy. **b**, 3D trajectory of the first trail by the medical intern with teleoperation (i.e. without AI co-pilot). **c**, 3D trajectory of the second trail by the medical intern with teleoperation. **d**, 3D trajectory of the third trail by the medical intern with teleoperation. **e**, 3D trajectory by the expert doctor with teleoperation. **f**, 3D trajectory by the attending doctor with AI co-pilot.

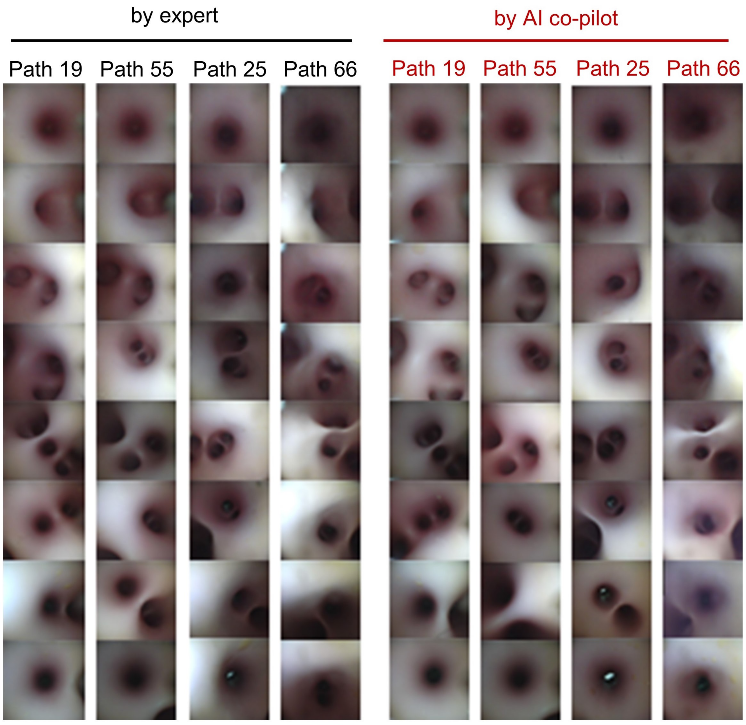

Supplementary Fig. 14. Images on multiple paths obtained by the paticipents. In the experiment, the bronchoscope robot could successfully be controlled to deep into the bronchial phantom along the specified paths, with the cavities centrally located in each endoscopic image.

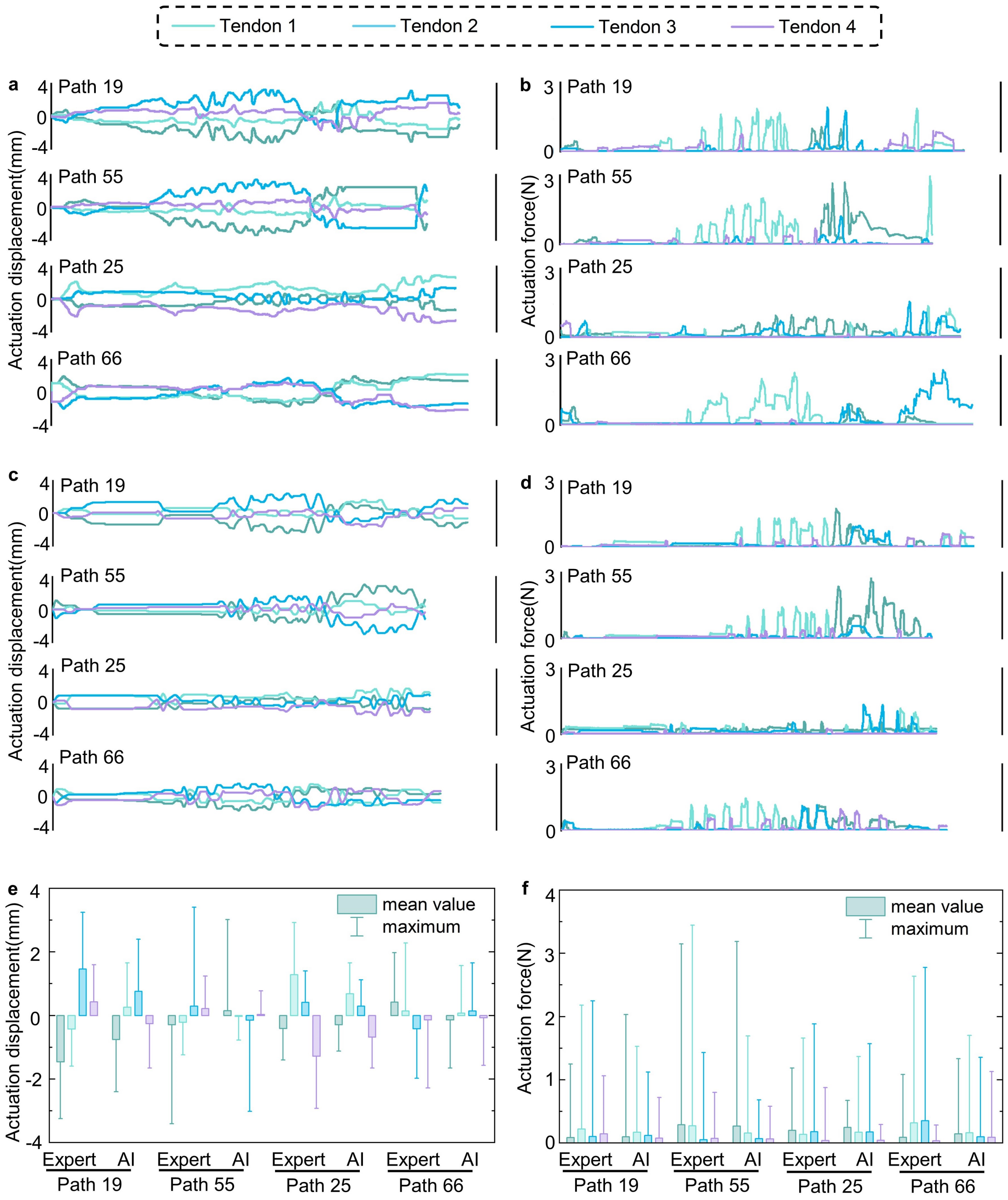

Supplementary Fig. 15. Actuation information on multiple paths obtained by the paticipents. a, Actuation displacement curves obtained by the expert with teleoperation on two phantoms. **b**, Actuation force curves obtained by the expert on two phantoms. **c**, Actuation displacement curves obtained by the attending doctor with AI co-pilot on two phantoms. **d**, Actuation force curves obtained by the attending doctor with AI co-pilot on two phantoms. **e**, Distribution of actuation displacements obtained by the expert and the attending doctor with AI co-pilot. The numbers of recorded points are n = 3023, 2787, 2997, 3088, 3086, 2772, 2806 and 2885 for eight independent experiments. **f**, Distribution of actuation forces obtained by the expert and the attending doctor with AI co-pilot. The numbers of recorded points are n = 3023, 2787, 2997, 3088, 3086, 2772, 2806 and 2885 for eight independent experiments.

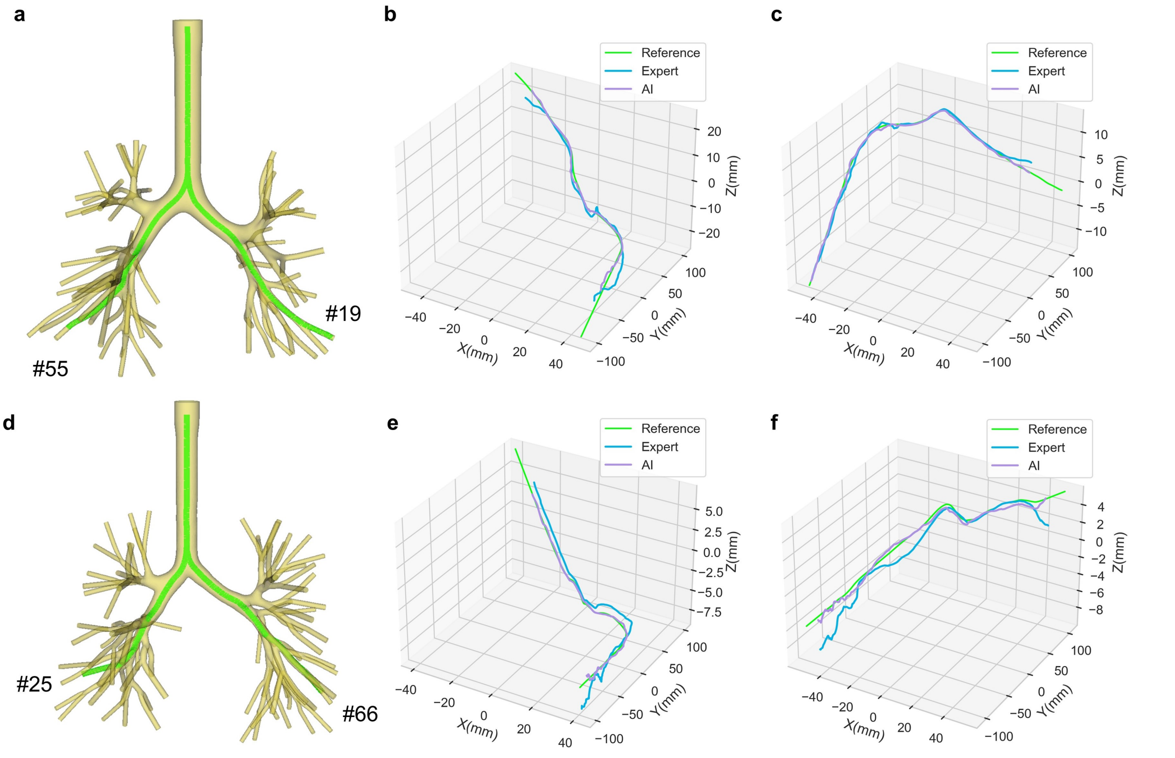

Supplementary Fig. 16. 3D trajectories on multiple paths obtained by the paticipents. a, The airway model of Phantom 1. Two paths are selected for bronchoscopy. Some key frames around the tracheal carina are used to align the time stamps of AI and expert groups with that of reference paths for showing 3D trajectories. **b**, Results of Path-55. **c**, Results of Path-19. **d**, Results of the airway model of Phantom 2. **e**, Results of Path-25. **f**, Results of Path-66. It's shown that 3D trajectories by AI co-pilot are closer to the reference path than that of the teleoperation.

Supplementary Note 9. Metrics

Success rate: In the simulation experiment, a reference path is defined as a centreline of the airway model from the start point of trachea to the end point of terminal bronchus. For example, a total of 60 reference paths are extracted in our testing airway model, shown as green lines in Supplementary Fig. 17a. A successful path is defined as follows: the simulated robot reaches the range within 1cm of the end point of the reference path, without any collision and wrong path choice during the running process, shown as the red line in Supplementary Fig. 17b. A collision is occurred if the distance between the robot and the inner wall is less than 0.1mm, as shown in Supplementary Fig. 17c. A wrong path choice is detected if the robot exceeds a virtual tunnel around the reference path, as shown in Supplementary Fig. 17d.

The success rate (SR) is defined as the ratio of successful path to all paths:

$$SR = N_s/N_{all} \tag{13}$$

Successful path ratio: For each reference path, the successful path ratio (SPR) is defined as the ratio of completed length to the total length of the reference path:

$$SPR_i = L_s^i/L_{all}^i \tag{14}$$

where L_s^i is the path length along reference path from the start point to the stopping point of simulated robot. If the robot successfully reaches the end point, the SPR_i will be set to 1. If robot fails in the process, the nearest waypoint on the reference path is regarded as the stopping point to calculate L_s^i.

The success rate can measure the generalization ability of the policy network to reach different branches of the bronchial tree and the successful path ratio can evaluate the coverage ability in the whole bronchial tree.

Image error: The accuracy of bronchoscopy procedures is commonly measured by the 3D position error, which is the Euclidean distance between the robot's head and the reference path (i.e. centreline) in 3D space, shown as e_{pos} in Supplementary Fig. 18a. Some existing works have reported position error results (also called absolute tracking error, ATE) of bronchoscopy in dead porcine lung or static phantom by aligning pre-operative reference path with realistic bronchial tree as the ground truth path. The key hypothesis of these works is that the lung is static and rigid when performing bronchoscopy. However, in clinical scenarios, due to the respiratory motion of live lung and the deformation of bronchus, all existing methods fails to accurately align reference paths with realistic bronchial tree, resulting in inaccurate 3D position error measurement, which is especially severe in deep bronchi. To address this issue, in this study, we use image error to measure the accuracy of bronchoscopy procedure, shown as e_{img} in Supplementary Fig. 18a. The image error is a widely used metric in the field of robot visual

serving, which measures the distance from image centre to the target position in image coordinate system without the need of 3D position. In practical terms, a mapping from e_{img} to e_{pos} can be formed in a statistical way, which we have performed new experiments for discussion in the following part.

The image error e_{img} is calculated by projecting the next direction vector $\hat{\mathbf{v}} \in \mathbb{R}^3$, predicted by the policy network, into the current image coordinate system of robot. In this study, we assume the policy network is well trained and $\hat{\mathbf{v}}$ should point to a waypoint lying on the centreline, satisfying

$$e_{img} = \|\overrightarrow{\mathbf{Op}}\| = \left\|\frac{1}{\hat{\mathbf{v}}|_z}\mathbf{K}\hat{\mathbf{v}}^T\right\| \tag{15}$$

where $\overrightarrow{\mathbf{Op}}$ is projected from $\hat{\mathbf{v}}$ in image, $\hat{\mathbf{v}}|_z$ is the z-coordinate value of $\hat{\mathbf{v}}$, $\mathbf{K} = \begin{bmatrix} f_x & 0 & 0 \\ 0 & f_y & 0 \end{bmatrix}$ is the known intrinsic matrix of camera, f_x and f_y are intrinsic parameters and satisfy $f_x = f_y$. Actually, $\hat{\mathbf{v}}$ is predicted by the policy network $\boldsymbol{\pi}$ and derived from the steering action $\mathbf{a} = \boldsymbol{\pi}(\mathbf{x}, c) = [\Delta\theta, \Delta\varphi]$. According to the formulation of ground truth steering action in Supplementary Fig. 18c, if the policy network is well trained, $\hat{\mathbf{v}}$ will direct to a point $\mathbf{P_a}$ on the centreline, and the projected $\overrightarrow{\mathbf{Op}}$ will direct to the bronchial lumen in image coordinate system. By using steering action, $\hat{\mathbf{v}}$ can be rotated from the current direction vector $\mathbf{v} = [0,0,d]$ of robot with a fixed depth d as

$$\hat{\mathbf{v}}^T = \mathbf{R}(\Delta\theta, \Delta\varphi)\mathbf{v}^T = \mathbf{R}(\Delta\theta, \Delta\varphi)\begin{bmatrix} 0 \\ 0 \\ d \end{bmatrix} \tag{16}$$

where $\mathbf{R}(\Delta\theta, \Delta\varphi) \in \mathbb{R}^{\mathbf{3}\times\mathbf{3}}$ is the rotation matrix from $\mathbf{v}$ to $\hat{\mathbf{v}}$, parameterized by the steering action $[\Delta\theta, \Delta\varphi]$ as

$$\mathbf{R}(\Delta\theta, \Delta\varphi) = \begin{bmatrix} \cos(\Delta\theta) & 0 & \sin(\Delta\theta) \\ \sin(\Delta\varphi)\sin(\Delta\theta) & \cos(\Delta\varphi) & -\cos(\Delta\theta)\sin(\Delta\varphi) \\ -\cos(\Delta\varphi)\sin(\Delta\theta) & \sin(\Delta\varphi) & \cos(\Delta\varphi)\cos(\Delta\theta) \end{bmatrix} \tag{17}$$

For simplicity, the rotation can also be parametrized by an angle $\psi = f(\Delta\theta, \Delta\varphi)$ about an axis of rotation, as shown in Supplementary Fig. 18a. Thus, we can rewrite Eq. 17 as following

$$e_{img} = \left\|\frac{1}{\hat{\mathbf{v}}|_z}\mathbf{K}\mathbf{R}(\Delta\theta, \Delta\varphi)\begin{bmatrix} 0 \\ 0 \\ d \end{bmatrix}\right\| = \frac{f_x d\sin(\psi)}{d\cos(\psi)} = f_x\tan(\psi) \tag{18}$$

If we assume that robot's head is parallel to the reference path (i.e. centreline) and the curve of centreline is small enough, the 3D position error e_{pos} can be approximated as

$$e_{pos} \approx d_a \tan(\psi) \tag{19}$$

where d_a is a fixed length along the centreline from the nearest waypoint to a far waypoint $\mathbf{P_a}$ pointed by $\hat{\mathbf{v}}$, as shown in Supplementary Fig. 18a. Thus, an approximate relation between e_{img} and e_{pos} can be obtained as

$$e_{img} = \frac{f_x}{d_a} d_a \tan(\psi) \approx \frac{f_x}{d_a} e_{pos} \propto e_{pos} \tag{20}$$

According to Eq. 20, it can be observed that e_{pos} has positive relation with e_{img}. However, in realistic bronchoscopy procedures, the two hypotheses (i.e., robot is parallel to airway centreline, and the centreline has small curve) are not always satisfied. Thus, we calibrate the pixel-to-millimeter conversion ratio $\Delta e = e_{pos}/e_{img}$ in a statistical way, generating a look-up figure as a reference which records Δe of every position in bronchus.

We randomly sample 13620 positions along four reference paths of two airway models in the virtual environment, as shown in Supplementary Fig. 18c and d. For each sample, the position and direction of robot head is randomly posed around the reference path and the image error e_{img} is calculated by policy network prediction and camera projection as the above process. The position error e_{pos} is measured by the Euclidean distance between robot's head and the reference path, which can be easily accessed in virtual environment. Then the pixel-to-millimeter conversion ratio Δe is calculated for each sample. The statistical results of Δe are shown in Supplementary Fig. 18b, and the mean results at every position of reference path are overlaid on Phantom 1 and 2, as displayed in Supplementary Fig. 18e, g and Supplementary Fig. 18f, h, demonstrating a decrease of Δe with the increase of bronchial generation. It's reasonable for these results because with the bronchoscope gradually going deeper, the bronchus becomes narrower and the e_{pos} becomes smaller, while e_{img} is only determined by robot's direction, so that Δe is smaller. Thus, according to Supplementary Fig. 18b, Δe is 0.075mm/pixel in in trachea (0th generation) and 0.018mm/pixel in 9th generation of bronchi, thus, 50-pixel image error means approximate 3.75mm and 0.91mm in 0th an 9th generation of bronchi, respectively. By averaging all samples in Supplementary Fig. 18b, the mean Δe is 0.064mm/pixel. In our in-vivo animal experiment, AI co-pilot group has a mean image error of 11.38 pixels, which reflects a mean 3D positon error of 0.73mm in whole procedures.

Human intervention ratio: We define human intervention as the number of switching actions of doctor's hand. Specifically, for teleoperation mode without AI assistance, we record the number of time stamps where the tendon displacements of linear motors change from last time stamp as the number of human interventions. For AI shared control mode, we record the number

of time stamps where discrete human command (e.g. up, down, left, right or forward) changes from last time stamp as the number of human interventions. The human intervention ratio (HIR) is defined as the ratio of the number of human interventions T_{interv} to the total number of time stamps T_{total} recorded during the whole bronchoscopy procedure:

$$HIR = T_{interv}/T_{total} \tag{21}$$

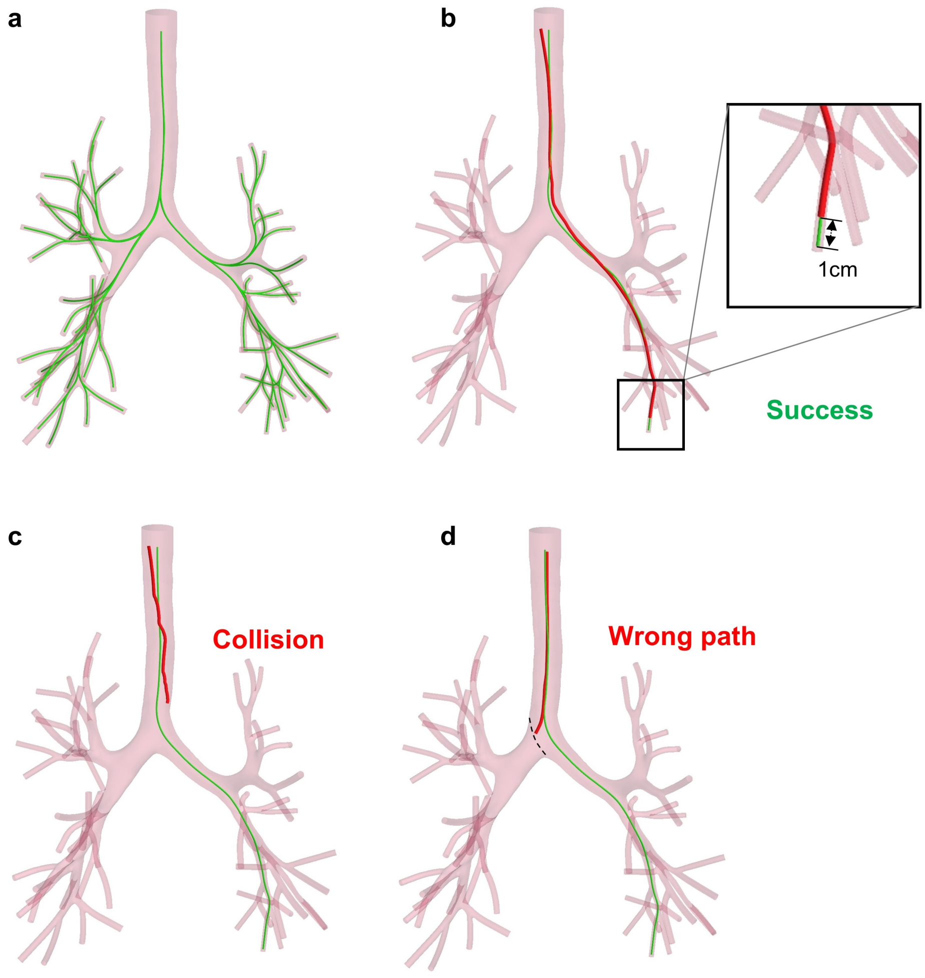

Supplementary Fig. 17. Reference paths and criteria of successful path. a, Reference paths (i.e., centrelines) of patient 3 for testing. **b**, An example of successful path that simulated robot reaches within a range of 1cm from the end point of the reference path. **c**, An example of failed path where the collision occurs between simulated robot and the inner bronchial wall. **d**, An example of failed path where the simulated robot enters into a wrong lumen.

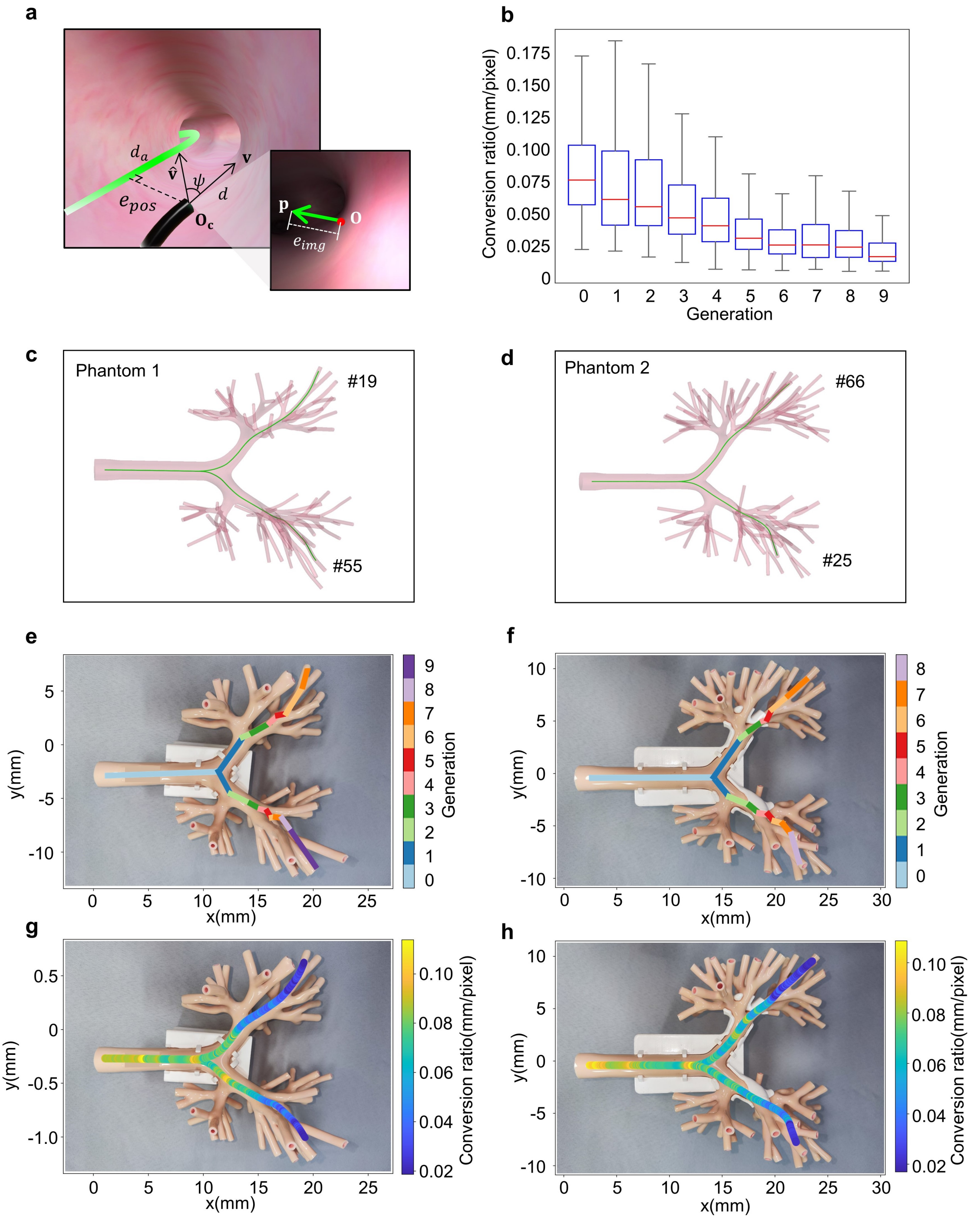

Supplementary Fig. 18. Image error to 3D position error mapping. a, Definitions of image error and 3D position error. The image error e_{img} is projected by a predicted direction vector $\hat{\mathbf{v}}$ by the policy network, which directs to a point lying on the centreline in 3D space. And the 3D position error e_{pos} is defined as the nearest distance between robot's head and the reference path. **b**, Statistical results of the pixel-to-millimeter conversion ratio in different bronchial generations. The number of sampled positions is n = 13620 along four reference paths of two airway models in the virtual environment. Red lines indicate median, box edges are 25th and 75th percentiles, whiskers indicate range. **c**, Virtual environment established on the airway model of Phantom 1 with its two reference paths Path 19 and 55. **d**, Virtual environment based on Phantom 2 airway model with Path 25 and 66. **e**, Segments of bronchial generations along Path 19 and 55 in Phantom 1. **f**, Segments of bronchial generations along Path 25 and 66 in Phantom 2. **g**, Distribution of pixel-to-millimeter conversion ratio along reference paths in

Phantom 1. **h**, Distribution of pixel-to-millimeter conversion ratio along reference paths in Phantom 2. It's obvious that with the bronchoscope robot reaching deep bronchus, the pixel-to-millimeter conversion ratio becomes smaller, because the diameter of airway tree becomes thinner.

Supplementary Note 10. NASA Task Load Index experiment

A novice doctor that has a little experience on robotic bronchoscopy and an expert doctor that has a lot experience are invited in this survey. Each doctor is asked to perform three trails of robotic bronchoscopy with and without AI co-pilot respectively. After every attempt of bronchoscopy, doctors are requested to complete a NASA TLX questionnaire.

The NASA TLX evaluates the perceived workload of humans across six subjective subscales. These subscales include mental demand (the level of mental exertion required by the task), physical demand (the level of physical effort required by the task), temporal demand (the sense of urgency or pace associated with the task), performance (the degree of success achieved in completing the task), effort (the amount of exertion needed to accomplish the performance level), and frustration (the extent of negative emotions such as insecurity, discouragement, irritation, stress, or annoyance experienced during the task). Each subscale is rated on a scale from 0 (very low) to 100 (very high), except for performance, which ranges from 0 (perfect) to 100 (failure). The comparison results between complete human control and AI co-pilot are depicted as Supplementary Fig. 19, demonstrating that the AI co-pilot bronchoscope robot can significantly reduce the physical fatigue and cognitive strain of users.

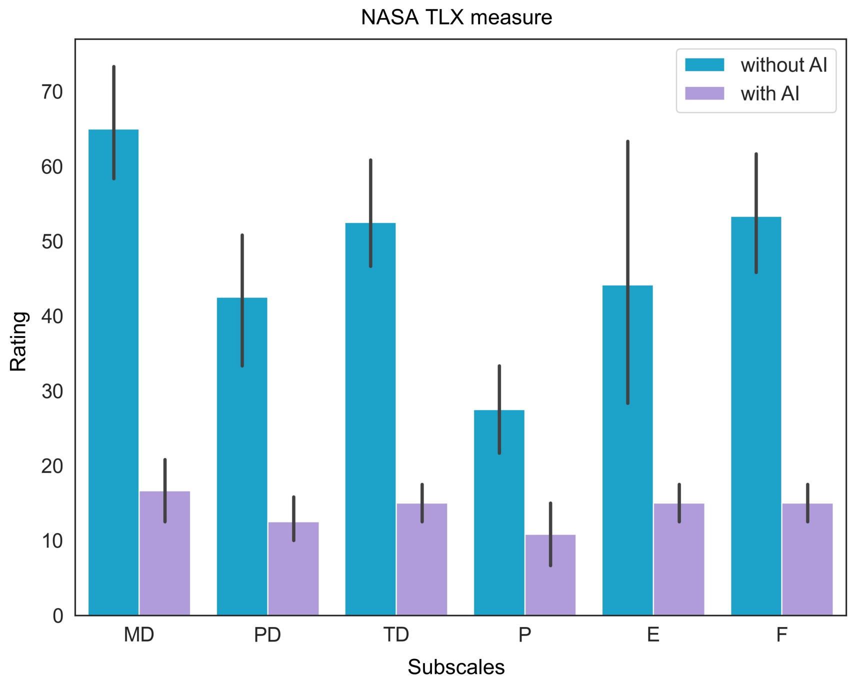

Supplementary Fig. 19. Multi-dimensional reports with and without AI co-pilot acquired by NASA-TLX questionnaires. It contains six subjective subscales, i.e., mental demand (MD), physical demand (PD), temporal demand (TD), performance (P), effort (E) and frustration (F). The smaller rating represents less user task load. A novice doctor and an expert doctor are invited in this survey. Each doctor is asked to perform n = 3 trails of robotic bronchoscopy with and without AI co-pilot respectively. The error bar denotes the 95% confidence intervals.

Supplementary Note 11. AI intervention and safety ensurement

In practice, AI co-pilot not only predicting the steering actions and displaying visual feedback to the surgeon, but also applying intervention and modifying the steering actions of surgeons. As shown in Supplementary Fig. 22, at first, we put the robot head close to the bronchial wall by teleoperation without AI co-pilot and keep doctor's hand still for no external control on robot. Then we start the AI co-pilot and doctor's hand also stay still. It's obvious that the image error decreases rapidly and automatically as the AI co-pilot control is triggered.

Doctors can take over full control of the robot at any time from the AI assistance if they deem it necessary. We have designed an interface for doctors to take control in our software-controlled GUI interface, as depicted in Supplementary Fig. 23. If the AI stop button is pressed, the AI-assisted function is disabled, and instead, the doctor can operate the robot manually through remote control. For safety purposes, our motor terminals are equipped with force sensors that can measure the tension of the wire. If the tension exceeds x newtons, our robot will automatically release the tension, allowing the end effector to lose rigidity and protect it from scraping against the inner wall of the airway.

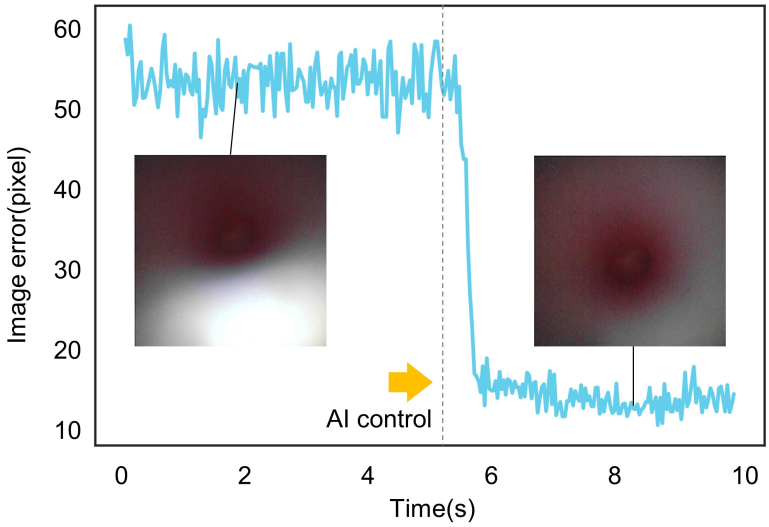

Supplementary Fig. 20. Visualisation of AI co-pilot intervention on human control.

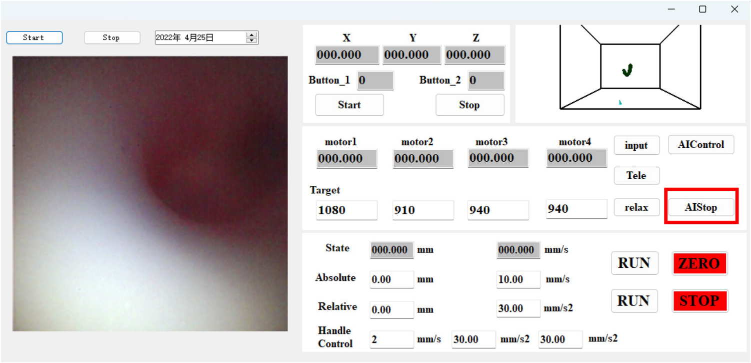

Supplementary Fig. 21. Software-controlled GUI interface.

Supplementary Note 12. AI control performance analysis

In order to analyze the overshooting and its influence on safety, we supplemented an experiment as depicted in Supplementary Fig. 23a. The robot catheter was controlled in advance to one side of the bronchial inner wall. Under AI control, the robot points towards the lumen centre, and in this process, we analyze the robot's motion trajectory by recording image error. As can be seen in Supplementary Fig. 23b, our robot hardly ever overshoots.

On the other hand, when bronchoscopy is performed, due to the interaction of respiratory movement and the movement of the robot, the tracking target of the robot's head is constantly changing, which requires the robot to track the target in real time. The overshoot shown in the video is mainly the error when tracking dynamic targets. As can be seen from the experimental results, compared with teleoperation, AI control still has good tracking accuracy, which proves its safety.

The use of only five discrete commands does not limit the motion of the robot to two planes and the robot can be bent with multiple tendons being pulled at a time. As shown in Supplementary Fig. 24, in a process of bronchoscopy, when the discrete human command is constantly “right”, the steering actions predicted by the policy network are not limited in horizontal plane but can be any direction according to the position of bronchial lumen in bronchoscopic image and the human command. For example, at t=0.05s, according to the human command “right”, the policy network identifies doctor’s intention of truing right and outputs a steering action that points to the centre of right bronchial lumen. At t=0.45s, the policy network keeps steering the robot to the right bronchial lumen. At t=1.05s, the robot enters into the right lumen and the policy network outputs a slightly left steering action for collision avoidance, although the human command is still “right”, leading to a safe bronchoscopy procedure. As a result, although the human commands are discrete, the steering actions predicted by the policy network are continuous and not limited to two planes, which are finally converted to continuous and safe tendon displacements for robot control.

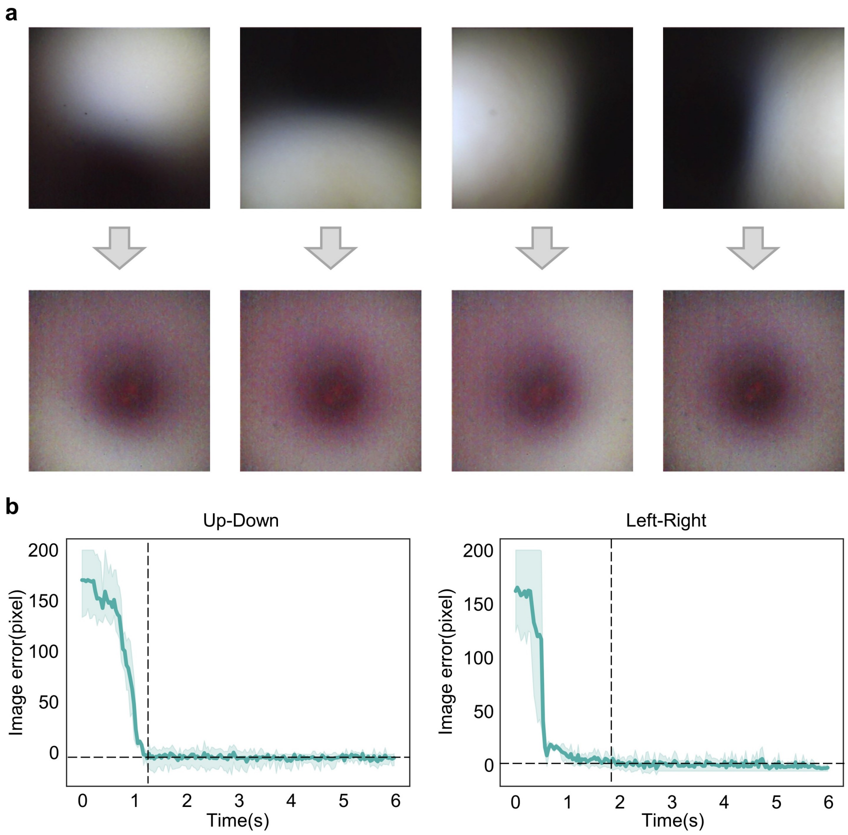

Supplementary Fig. 22. Closed-loop control experiment of the AI co-pilot bronchoscope robot.

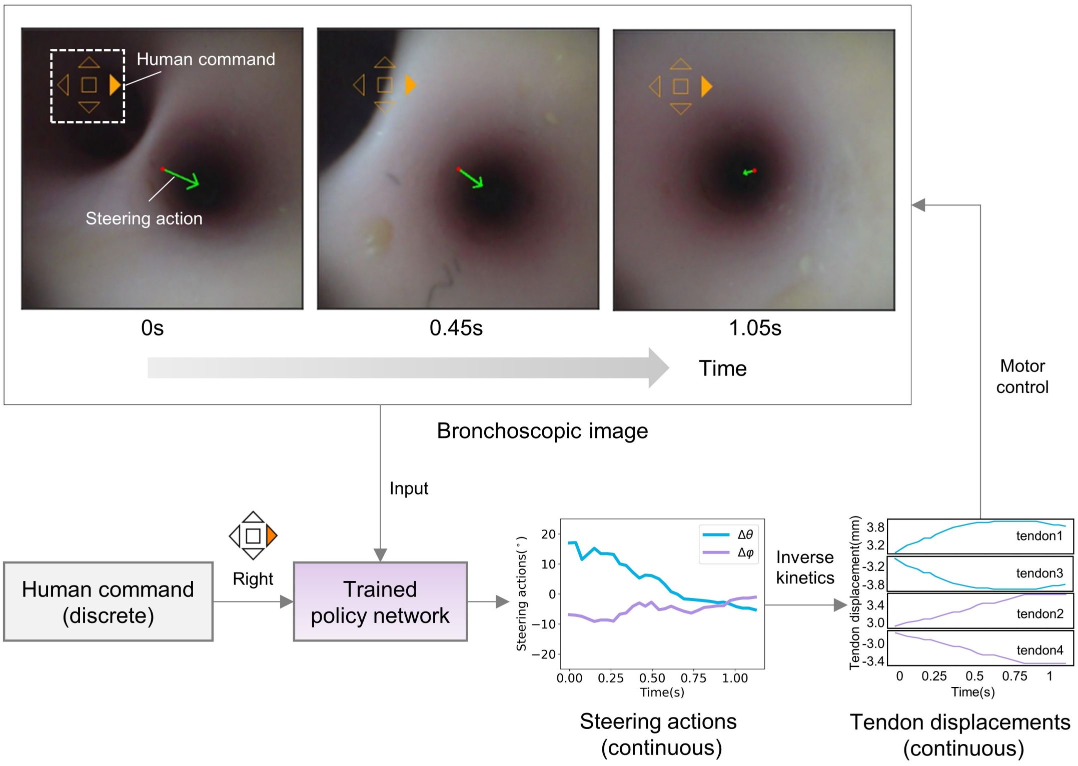

Supplementary Fig. 23. Visualization of steering actions and tendon displacements during bronchoscopy.

Supplementary Note 13. In-vivo animal experiment

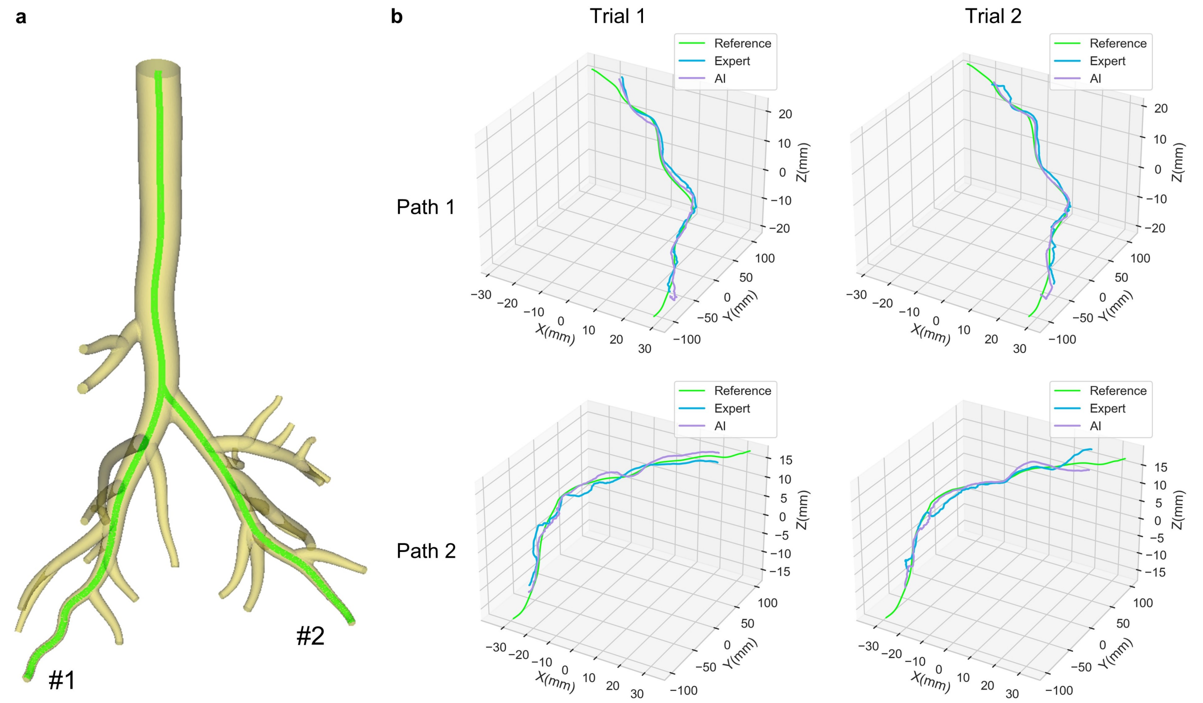

Supplementary Fig. 24. 3D trajectories of in-vivo experiments. a, The airway model of pig. Two paths are selected for bronchoscopy. **b**, 3D trajectories of two trails obtained by the expert and the attending doctor with AI co-pilot. It's demonstrated that the operation accuracy of the attending doctor with AI co-pilot is higher than that of expert's teleoperation.

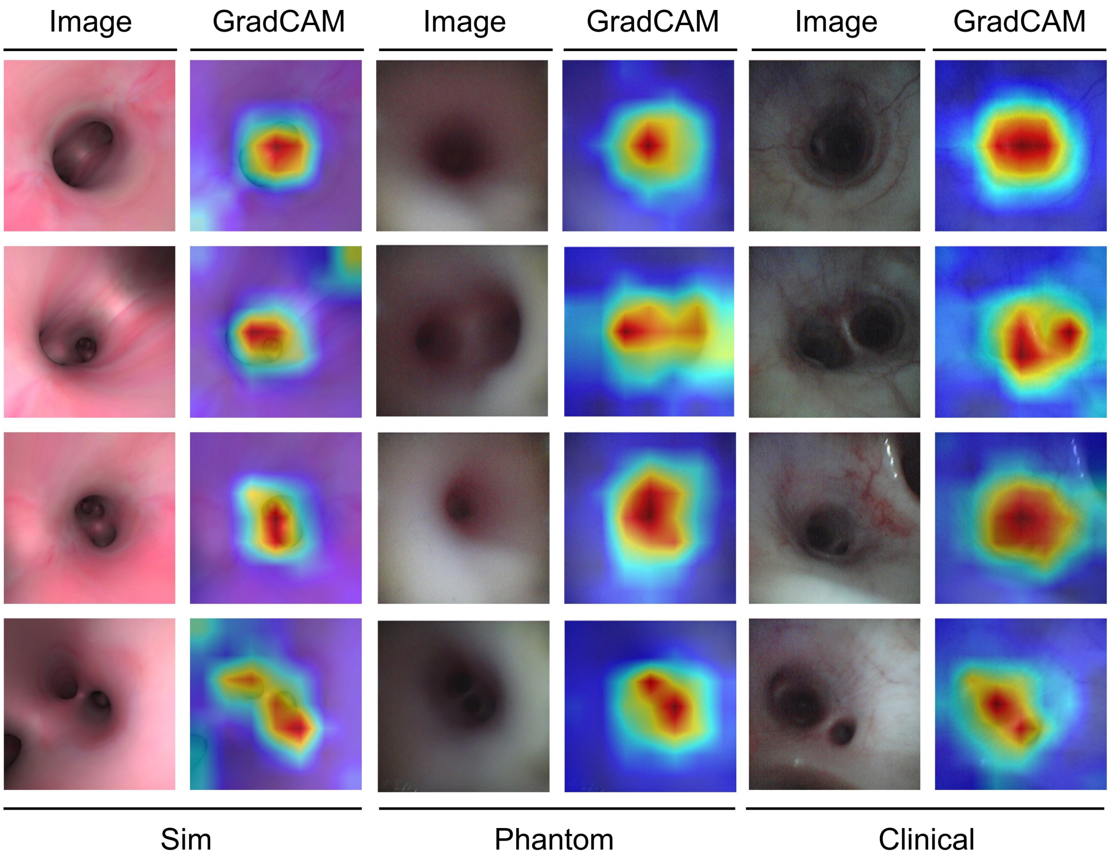

Supplementary Fig. 25. Gradient-weighted Class activation maps (GradCAM) from the last convolutional layer of our policy network. Three different styles of bronchoscopic images are chosen for validation.

Supplementary Table 1. The operation experience of participants in the experiments. The medical intern (novice 1), the attending doctor (novice 2) and the expert doctor (expert) both come from the School of Medicine at Zhejiang University, Hangzhou, China.

Participant	Professional title	Entire period of operation	Manual operations	Robot teleoperations
novice 1	medical intern	no	no	2 demonstrations
novice 2	attending doctor	<5 years	<100 cases per year	2 demonstrations
expert	chief doctor	>20 years	>200 cases per year	>100 trials

Supplementary Table 2. Robot costing. The standard version of the system without force sensors has the cost of $4168, while the advanced version of the system with force sensors has the cost of $13476.

**Equipment or mechanical parts**	**Model**	**unit price**	**num ber**	**total price**	**remarks**
linear motor	LA-50	$273	4	$1,092	used for steering the catheter
electric slide	EZSM3E040AZMK	$861	1	$861	used for feeding the catheter
slide drive module	AZD-KD	$350	1	$350	used for driving the electric slide
micro camera	OCHTA10	$525	1	$525	used for endoscopic imaging
snake tube	—	$410	1	$410	used to make the distal section
braided mesh tube	—	$410	1	$410	used to make the proximal section
waterproof rubber	—	$110	1	$110	used as a waterproof layer
machined parts	—	$410	1	$410	used as connecting parts or shells
force sensor*	qla414	$2,327	4	$9,308	used for force limit
**Standard version price**		**$4,168**	**Advanced version price**		**$13,476**

"*" means optional. The device be eliminated or substituted.

Supplementary Table 3. Network architecture of our domain adaptation method. The architecture includes a generator, discriminator and depth estimator. Conv represents the convolutional layer and Upconv denotes the transposed convolutional layer. RN is the basic ResNet block consisting of two convolutional layers with residual connection. Max pool represents max pooling layer. G_c and G_a share the same representation extracted by G_e. In depth estimator, some skip connections between convolutional and transposed convolutional layers are implemented.

Domain adaptation network structure

Generator

Network	Layer		Channels In	Channels Out	Kernel	Stride	Padding	Normalize	Activation
Ge	Conv1		3	64	7×7	1	0	Instance	ReLu
	Conv2		64	128	3×3	2	1	Instance	ReLu
	Conv3		128	256	3×3	2	1	Instance	ReLu
	Convx×9	RN_1	256	256	3×3	1	1	Instance	ReLu
		RN_2	256	256	3×3	1	1	Instance	None
Gc	UpConv1		256	128	3×3	2	1	Instance	ReLu
	UpConv2		128	64	3×3	2	1	Instance	ReLu
	Conv13		64	27	7×7	1	0	None	Tanh
Ga	UpConv1		256	128	3×3	2	1	Instance	ReLu
	UpConv2		128	64	3×3	2	1	Instance	ReLu
	Conv14		64	10	1×1	1	0	None	Softmax

Depth estimator

Layer		Channels In	Channels Out	Kernel	Stride	Padding	Normalize	Activation
Conv15		3	64	7×7	2	3	Batch	ReLu
Max pool		64	64	3×3	2	1	None	None
Convx×2	RN_1	64	64	3×3	1	1	Batch	ReLu
	RN_2	64	64	3×3	1	1	Batch	ReLu
Convx×4	RN_1	128	128	3×3	2	1	Batch	ReLu
	RN_2	128	128	3×3	2	1	Batch	ReLu
Convx×6	RN_1	256	256	3×3	2	1	Batch	ReLu
	RN_2	256	256	3×3	2	1	Batch	ReLu
Convx×3	RN_1	512	512	3×3	2	1	Batch	ReLu
	RN_2	512	512	3×3	2	1	Batch	ReLu
Upconv3	Upconv	512	256	2×2	2	0	Batch	ReLu
	RN_1	512	256	3×3	1	1	Batch	ReLu
	RN_2	256	256	3×3	1	1	Batch	ReLu
Upconv4	Upconv	256	128	2×2	2	0	Batch	ReLu
	RN_1	256	128	3×3	1	1	Batch	ReLu
	RN_2	128	128	3×3	1	1	Batch	ReLu
Upconv5	Upconv	128	64	2×2	2	0	Batch	ReLu
	RN_1	128	64	3×3	1	1	Batch	ReLu
	RN_2	64	64	3×3	1	1	Batch	ReLu
Upconv6	RN_1	128	64	3×3	1	1	Batch	ReLu
	RN_2	64	64	3×3	1	1	Batch	ReLu
Upconv7		64	32	2×2	2	0	None	None
Conv16		32	1	1×1	1	0	None	None

Discriminator

Layer	Channels In	Channels Out	Kernel	Stride	Padding	Normalize	Activation
Conv1	3	64	4×4	2	1	None	LeakyReLu
Conv2	64	128	4×4	2	1	Instance	LeakyReLu
Conv3	128	256	4×4	2	1	Instance	LeakyReLu
Conv4	256	512	4×4	1	1	Instance	LeakyReLu
Conv5	512	1	4×4	1	1	None	None

Supplementary Table 4. Policy network architecture. Conv represents the convolutional layer and Upconv denotes the transposed convolutional layer. RN is the basic ResNet block consisting of two convolutional layers with residual connection. FC is the fully connected layer (or multilayer perceptron, MLP). Max pool and Avg pool represent max and average pooling layer, respectively. Five action heads and the depth decoder share the same representation extracted by the feature extractor. Some skip connections between the feature extractor and depth decoder are also implemented.

Policy network structure

Network	Layer		Channels In	Channels Out	Kernel	Stride	Padding	Normalize	Activation
Feature extractor	Conv15		3	64	7×7	2	3	Batch	ReLu
	Max pool		64	64	3×3	2	1	None	None
	Convx×2	RN_1	64	64	3×3	1	1	Batch	ReLu
		RN_2	64	64	3×3	1	1	Batch	ReLu
	Convx×4	RN_1	128	128	3×3	2	1	Batch	ReLu
		RN_2	128	128	3×3	2	1	Batch	ReLu
	Convx×6	RN_1	256	256	3×3	2	1	Batch	ReLu
		RN_2	256	256	3×3	2	1	Batch	ReLu
	Convx×3	RN_1	512	512	3×3	2	1	Batch	ReLu
		RN_2	512	512	3×3	2	1	Batch	ReLu
	Avg pool		512	512	None	None	None	None	None
	FC		512	512	None	None	None	None	None
Action head	FC1		512	512	None	None	None	None	ReLu
	FC2		512	256	None	None	None	None	ReLu
	FC3		256	2	None	None	None	None	Tanh
Depth decoder	Upconv3	Upconv	512	256	2×2	2	0	Batch	ReLu
		RN_1	512	256	3×3	1	1	Batch	ReLu
		RN_2	256	256	3×3	1	1	Batch	ReLu
	Upconv4	Upconv	256	128	2×2	2	0	Batch	ReLu
		RN_1	256	128	3×3	1	1	Batch	ReLu
		RN_2	128	128	3×3	1	1	Batch	ReLu
	Upconv5	Upconv	128	64	2×2	2	0	Batch	ReLu
		RN_1	128	64	3×3	1	1	Batch	ReLu
		RN_2	64	64	3×3	1	1	Batch	ReLu
	Upconv6	RN_1	128	64	3×3	1	1	Batch	ReLu
		RN_2	64	64	3×3	1	1	Batch	ReLu
	Upconv7		64	32	2×2	2	0	None	None
	Conv16		32	1	1×1	1	0	None	None
