# Supplementary material for: AI co-pilot bronchoscope robot
Source: Nat Commun. 2024 Jan 4;15:241. doi: 10.1038/s41467-023-44385-7 (PMC10764930; doi:10.1038/s41467-023-44385-7)
Supplement: Supplementary file 3 — Description of Additional Supplementary Files [file 41467_2023_44385_MOESM3_ESM.pdf]

**Description of Additional Supplementary Files**

**File name: Supplementary Movie 1**
**Description: Overview of AI co-pilot bronchoscope robot.**
The movie introduces the design of AI co-pilot bronchoscope robot, the component of AI-human shared control algorithm, teleoperation experiment, simulation results, self-centering control experiments, in vitro and in vivo experiments.

**File name: Supplementary Movie 2**
**Description: In Vitro Experiment of Bronchial Phantom 1.**
The movie shows the bronchoscopy procedures by the expert with teleoperation and the novice doctor with AI co-pilot on the bronchial phantom 1.

**File name: Supplementary Movie 3**
**Description: In Vitro Experiment of Bronchial Phantom 2.**
The movie shows the bronchoscopy procedures by the expert with teleoperation and the novice doctor with AI co-pilot on the bronchial phantom 2.

**File name: Supplementary Movie 4**
**Description: In Vivo Experiment of Live Porcine Lung Model**
The movie shows the bronchoscopy procedure by the expert with teleoperation and the novice doctor with AI co-pilot in the live porcine lung.
