# Supplementary material for: AI co-pilot bronchoscope robot
Source: Nat Commun. 2024 Jan 4;15:241. doi: 10.1038/s41467-023-44385-7 (PMC10764930; doi:10.1038/s41467-023-44385-7)
Supplement: Supplementary file 8 — Reporting Summary [file 41467_2023_44385_MOESM8_ESM.pdf]

nature portfolio

Corresponding author(s): Rong Xiong, Yue Wang, Haojian Lu

Last updated by author(s): Sep 30, 2023

Reporting Summary

Nature Portfolio wishes to improve the reproducibility of the work that we publish. This form provides structure for consistency and transparency in reporting. For further information on Nature Portfolio policies, see our Editorial Policies and the Editorial Policy Checklist.

Statistics

For all statistical analyses, confirm that the following items are present in the figure legend, table legend, main text, or Methods section.

n/a	Confirmed	
☐	☒	The exact sample size (*n*) for each experimental group/condition, given as a discrete number and unit of measurement
☐	☒	A statement on whether measurements were taken from distinct samples or whether the same sample was measured repeatedly
☒	☐	The statistical test(s) used AND whether they are one- or two-sided<br>*Only common tests should be described solely by name; describe more complex techniques in the Methods section.*
☒	☐	A description of all covariates tested
☒	☐	A description of any assumptions or corrections, such as tests of normality and adjustment for multiple comparisons
☐	☒	A full description of the statistical parameters including central tendency (e.g. means) or other basic estimates (e.g. regression coefficient) AND variation (e.g. standard deviation) or associated estimates of uncertainty (e.g. confidence intervals)
☒	☐	For null hypothesis testing, the test statistic (e.g. *F*, *t*, *r*) with confidence intervals, effect sizes, degrees of freedom and *P* value noted<br>*Give P values as exact values whenever suitable.*
☒	☐	For Bayesian analysis, information on the choice of priors and Markov chain Monte Carlo settings
☐	☒	For hierarchical and complex designs, identification of the appropriate level for tests and full reporting of outcomes
☒	☐	Estimates of effect sizes (e.g. Cohen's *d*, Pearson's *r*), indicating how they were calculated

Our web collection on statistics for biologists contains articles on many of the points above.

Software and code

Policy information about availability of computer code

Data collection	The AI-human shared control algorithm is implemented using Python (v3.7.11). The virtual environment for visualization, robot simulation and human commands generation for control is Pybullet (v3.2.2). The virtual bronchoscopic images and depths are rendered and collected by Pyrender (v0.1.45). To establish the virtual bronchoscopy environment, the airway models are segmented from CT scans using 3D Slicer (v4.10.2). The airway centrelines are then extracted using VMTK (v1.4.0).
Data analysis	The data processing tools include NumPy (v1.19.5), OpenCV (v4.5.5.64) and VTK (v8.2.0). The data analysis and visualization are implemented using Matplotlib (v3.3.4) and MATLAB R2022a. The policy network and Sim2Real adaptation module are implemented using the PyTorch platform (v1.9.1) and Python (v3.7.11). The benchmark method is AttentionGAN (https://github.com/Ha0Tang/AttentionGAN) and CycleGAN (https://github.com/junyanz/pytorch-CycleGAN-and-pix2pix). The code of our AI-human shared control algorithm is available on GitHub (https://github.com/LiuLiluZJU/AI-Co-Pilot-Bronchoscope-Robot). The DOI for the code is https://doi.org/10.5281/zenodo.10077315. The repository includes virtual environment establishment, data acquisition, image processing, visualization, network training and testing code.

For manuscripts utilizing custom algorithms or software that are central to the research but not yet described in published literature, software must be made available to editors and reviewers. We strongly encourage code deposition in a community repository (e.g. GitHub). See the Nature Portfolio guidelines for submitting code & software for further information.

Data

Policy information about availability of data

All manuscripts must include a data availability statement. This statement should provide the following information, where applicable:

- Accession codes, unique identifiers, or web links for publicly available datasets
- A description of any restrictions on data availability
- For clinical datasets or third party data, please ensure that the statement adheres to our policy

The dataset of this paper has been uploaded to Zenodo for public availability, including the data for establishing a virtual bronchoscopy environment for network training and testing, as well as the trained network model for evaluating our conclusions. The links to the two parts of the dataset are as follows: virtual bronchoscopy environment data (https://zenodo.org/records/10077275) and the trained network model data (https://zenodo.org/records/10077290). This study does not include clinical datasets or data from third parties.

Research involving human participants, their data, or biological material

Policy information about studies with human participants or human data. See also policy information about sex, gender (identity/presentation), and sexual orientation and race, ethnicity and racism.

Reporting on sex and gender	N/A
Reporting on race, ethnicity, or other socially relevant groupings	N/A
Population characteristics	N/A
Recruitment	N/A
Ethics oversight	This paper does not involve the research of human participants.

Note that full information on the approval of the study protocol must also be provided in the manuscript.

Field-specific reporting

Please select the one below that is the best fit for your research. If you are not sure, read the appropriate sections before making your selection.

☒ Life sciences ☐ Behavioural & social sciences ☐ Ecological, evolutionary & environmental sciences

For a reference copy of the document with all sections, see nature.com/documents/nr-reporting-summary-flat.pdf

Life sciences study design

All studies must disclose on these points even when the disclosure is negative.

Sample size	Statistical methods were not used to determine sample size.The sample sizes used in our study are considered adequate and consistent with the literature in the field, for number of airway models, number of bronchoscopy trials and number of training samples. (E.g. PMID: 32047155, PMID: 23508260, PMID: 33089071, PMID: 35839186, PMID: 34314953, PMID: 37729421).
Data exclusions	No data were excluded from the analyses.
Replication	All experiments were repeated at least three times. All attempts at replication were successful.
Randomization	The choice of intubation path was determined by randomization. When conducting other experiments, randomization or all-test strategies are also used to test the performance of the robot.
Blinding	Blinding was not relevant to our study because the all the experiments were aiming to verify the validity of AI co-pilot bronchoscope robot system rather than for comparison of finding.

Reporting for specific materials, systems and methods

We require information from authors about some types of materials, experimental systems and methods used in many studies. Here, indicate whether each material, system or method listed is relevant to your study. If you are not sure if a list item applies to your research, read the appropriate section before selecting a response.

Materials & experimental systems

n/a	Involved in the study
☒	☐ Antibodies
☒	☐ Eukaryotic cell lines
☒	☐ Palaeontology and archaeology
☐	☒ Animals and other organisms
☒	☐ Clinical data
☒	☐ Dual use research of concern
☒	☐ Plants

Methods

n/a	Involved in the study
☒	☐ ChIP-seq
☒	☐ Flow cytometry
☒	☐ MRI-based neuroimaging

Animals and other research organisms

Policy information about studies involving animals; ARRIVE guidelines recommended for reporting animal research, and Sex and Gender in Research

Laboratory animals	The in-vivo experiment used a pig purchased from Zhuhai BesTest Bio-Tech Co.,Ltd. It was 1.2 meters long, weighed 39 kilograms and was in good health.
Wild animals	The study did not involve wild animals.
Reporting on sex	This pig is female.
Field-collected samples	The samples did not involve samples collected from the field.
Ethics oversight	The pig was purchased from Zhuhai BesTest Bio-Tech Co.,Ltd., solely based on the health condition. The protocols of animal experiments were approved by the Institutional Animal Care and Ethics Committee of Zhuhai BestTest (IAC(S)2201003-1).

Note that full information on the approval of the study protocol must also be provided in the manuscript.
